# First characterization of cultivable extremophile *Chroococcidiopsis* isolates from a solar panel

**DOI:** 10.3389/fmicb.2023.982422

**Published:** 2023-02-17

**Authors:** Sara Baldanta, Raquel Arnal, Amaya Blanco-Rivero, Govinda Guevara, Juana María Navarro Llorens

**Affiliations:** Metabolic Engineering Group, Department of Biochemistry and Molecular Biology, Universidad Complutense de Madrid, Madrid, Spain

**Keywords:** extremophile cyanobacteria, solar panel, desiccation, UV-C, *Chroococcidiopsis*, SEVA vectors

## Abstract

**Introduction:**

Microorganisms colonize a wide range of natural and artificial environments. Even though most of them are unculturable in laboratory conditions, some ecosystems are ideal niches for bioprospecting extremophiles with unique properties. Up today, there are few reports concerning microbial communities found on solar panels, a widespread, artificial, extreme habitat. Microorganisms found in this habitat belong to drought-, heat- and radiation-adapted genera, including fungi, bacteria, and cyanobacteria.

**Methods:**

Here we isolated and identified several cyanobacteria from a solar panel. Then, some strains isolated were characterizated for their resistance to desiccation, UV-C exposition, and their growth on a range of temperature, pH, NaCl concentration or diverse carbon and nitrogen sources. Finally, gene transfer to these isolates was evaluated using several SEVA plasmids with different replicons to assess their potential in biotechnological applications.

**Results and discussion:**

This study presents the first identification and characterization of cultivable extremophile cyanobacteria from a solar panel in Valencia, Spain. The isolates are members of the genera *Chroococcidiopsis*, *Leptolyngbya*, *Myxacorys*, and *Oculatella* all genera with species commonly isolated from deserts and arid regions. Four of the isolates were selected, all of them *Chroococcidiopsis*, and characterized. Our results showed that all *Chroococcidiopsis* isolates chosen were resistant up to a year of desiccation, viable after exposition to high doses of UV-C, and capable of being transformed. Our findings revealed that a solar panel is a useful ecological niche in searching for extremophilic cyanobacteria to further study the desiccation and UV-tolerance mechanisms. We conclude that these cyanobacteria can be modified and exploited as candidates for biotechnological purposes, including astrobiology applications.

## 1. Introduction

Microorganisms colonize a wide variety of natural and artificial environments. Although some extreme environments are ideal niches for the bioprospection of extremophiles with unique properties for biotechnological applications, most of the microorganisms identified by metagenomics studies are uncultivable in ordinary laboratory conditions (Steen et al., 2019). One of these extreme environments of interest, and of which there are few reports of microbial communities living in them, are solar panels, prevalent, artificial, extreme habitats that are subject to different stresses, such as high irradiation, temperature fluctuations, and desiccation (Dorado-Morales et al., 2016; Porcar et al., 2018; Tanner et al., 2018). Recently, a two-year study has showed that solar panel surfaces can be colonized by microorganisms adapted to desiccation, temperature fluctuations and solar radiation in which species richness and biodiversity exhibit seasonal fluctuations forcing the selection of a particular microbial community (Tanner et al., 2020). The biocenosis tends to be formed by extremophilic bacterial genera *Deinococcus*, *Hymenobacter* and *Roseomonas* and the fungal genera *Neocatenulostroma*, *Symmetrospora*, and *Sporobolomyces* (Tanner et al., 2020). The taxonomic profiles derived from the metagenomic data of the solar panels in Berkeley and Valencia, which share a similar Mediterranean climate, demonstrated to be remarkably similar. The most abundant taxa were Actinobacteria (16%), Bacteroidetes (23%), Cyanobacteria (<3%), Deinococcus (6%), Firmicutes (<3%), Proteobacteria (15%), and Ascomycota (32%) (Porcar et al., 2018).

Cyanobacteria are among the oldest living organisms on the planet, and they are the only known prokaryotes capable of carrying out oxygenic photosynthesis. Cyanobacteria generally exhibit a high level of adaptive abilities and tolerance to many environmental stresses (Singh, 2018). They can be found in all ecosystems on Earth, from oceans, rivers, and freshwater lakes to hot springs and deserts (Boone and Castenholz, 2001; Azua-Bustos et al., 2022). Cyanobacteria are critical in maintaining soil stability and nutrient cycles and currently, they are used as an effective approach to restore habitats and accelerate ecosystem regeneration (Bowker, 2007; Zhang et al., 2016; Muñoz-Rojas et al., 2018; Muñoz-Martín et al., 2019; Zhao et al., 2019). Apart from their ecological role in global carbon and nitrogen cycles, they are also attractive as a promising source in biotechnological applications in various fields, such as biofuels and pharmaceuticals. Therefore, cyanobacterial diversity is important in terms of exploring and developing their potential for human benefit.

Members of the genus *Chroococcidiopsis* are widespread unicellular cyanobacteria that have been isolated from diverse extreme habitats: from hot deserts such as the Mojave in the southwestern part of the United States (Smith et al., 2014) or the temperate desert Atacama in Chile (Warren-Rhodes et al., 2007) to the cold Dry Valleys in Antarctica (Friedmann and Ocampo, 1976). But they have also been isolated from hypersaline environments (e.g., the sea or brackish water, Dor et al., 1991), Indonesian hot springs (Geitler, 1933), or porous rocks (Wierzchos et al., 2018; Bothe, 2019). A marked feature of *Chroococcidiopsis* is its high resistance to both desiccation and radiation (Billi et al., 2001; Singh, 2018). For instance, the desert strain *Chroococcidiopsis* sp. CCMEE 029 was able to grow after 4 years of air-drying under laboratory conditions (Fagliarone et al., 2017) and dried biofilms of that strain survived being exposed to space vacuum in low Earth orbit outside the International Space Station for 672 days (Mosca et al., 2021). When dried, other *Chroococcidiopsis* sp. strains tolerated up to 24 kGy of gamma rays (Verseux et al., 2017) and 1.5×10^3^ kJ m^−2^ of Mars-like UV flux (Mosca et al., 2019, 2021). The ability to survive in those harsh conditions has led to *Chroococcidiopsis* being a common genus in astrobiology studies and considered a suitable organism for the terraforming of Mars (Friedmann and Ocampo-Friedmann, 1995; Bothe, 2019). Moreover, gene transfer in some *Chroococcidiopsis* sp. strains *via* conjugation has been reported, and plasmids were maintained in the cells after 18 months of dry storage (Billi, 2012). In addition to their application in astrobiology, *Chroococcidiopsis* is preadapted to stresses that often correlate with industrial needs. Therefore, the strains could be particularly relevant for pharmacological, cosmetic, and food industries due to the production of pigments and singular metabolites, such as UV light absorbents or antioxidants (Montero-Lobato et al., 2020; Assunção et al., 2021). In the search for versatile biotechnological enzymes and the industrial implementation of bioprocesses, enzymes from extremophiles may be valuable biocatalysts that can endure different stresses. For instance, the bioinformatic analysis of the complete genome of *Chroococcidiopsis thermalis* PCC 7203 (NC_019695) has allowed to find and characterize a gene encoding a nucleoside 2′-deoxyribosyltransferase whose product is thermostable and acid tolerant (Del Arco et al., 2018).

In the present work, we have identified 10 cyanobacteria from a solar panel located in Valencia (Spain), an artificial outdoor environment exposed to cycles of low/high temperatures and constant hydration-dehydration periods with minimal water retention (Dorado-Morales et al., 2016). All the cyanobacteria isolated were phylogenetically close to those common in deserts and arid locations. Our work shows for the first time the characterization of culturable *Chroococcidiopsis* isolates from a solar panel in different laboratory growth conditions in a range of NaCl, pH, temperature, nitrogen, and carbon sources, but also displaying extremophile properties, e.g., long-term desiccation and UV-C resistance. Moreover, several plasmids, including CRISPR-Cas and expression systems were successfully transferred in several *Chroococcidiopsis* isolates, leaving the door open for its manipulation for different applications, including astrobiology research.

## 2. Materials and methods

### 2.1. Isolation and culture conditions

Sample collection was performed during the summer solstice of 2013 and 2014 in a set of solar panels situated in several locations in Valencia (Spain) by Dorado-Morales et al. (2016). Briefly, sterile PBS was poured on the panel and the liquid was harvested by scraping the surface with a sterile window cleaner. During the sampling process, the average temperature of the panels’ surfaces was 51°C and the average solar irradiance in Valencia was 461.3 W m^−2^. Samples were stored at −80°C until their use. Further detailed information about field collection and samples is provided in Dorado-Morales et al. (2016).

Standard methods were used for the isolation and cultivation of cyanobacteria. Samples were spread on plates (1.5% wt/vol Purified Agar) with different cyanobacterial media: BG11 (Rippka et al., 1979), *Spirulina* medium (hereafter UTEX, Starr and Zeikus, 1987), Castenholz-D medium (Mühlenhoff and Chauvat, 1996), and MDM (Watanabe, 1960; Supplementary Table S1). BG11 and MDM were also used without nitrogen combined sources (BG11_0_ and MDM_0_, respectively). As cyanobacteria from environmental samples often grow more slowly than other bacteria, growth was observed in parallel in media with and without 20 μg mL^−1^ cycloheximide, 10 μg mL^−1^ nystatin, and 15 μg mL^−1^ nalidixic acid added to prevent the growth of yeasts, fungi, other eukaryotic microorganisms, and fast-growing Gram-negative bacteria. The cultures were grown under continuous illumination (10 μmol photon m^−2^ s^−1^) at 25–30°C for about 1–2 months. Then, non-axenic isolated cyanobacteria were grown routinely in both solid and liquid media in continuous illumination of 60–80 μmol photon m^−2^ s^−1^. For some strains, cultures were maintained with rotary shaking (150 rpm) at 30°C and 100 μmol photon m^−2^ s^−1^. Pure cyanobacteria cultures of best-growing isolates were finally obtained by repeatedly streaking on agar-solidified BG11 supplemented with 5% (vol/vol) LB. When needed, light intensity was measured with a PCE-174 luxometer.

An axenic test for those cultures was carried out, as previously described (Taton et al., 2012) with slight modifications. Aliquots of 50 μL of grown isolates were inoculated into 4 different solid media: (1) BG11 with 5% LB (vol/vol), (2) BG11 with 5% LB (vol/vol) and 0.04% glucose (wt/vol), (3) BG11 with 0.01% glucose, 0.01% yeast extract (wt/vol), and 0.01% tryptone (wt/vol), and (4) LB. Plates were incubated at 30 or 37°C in darkness for 1 month. If no growth of heterotrophic bacteria was observed in the plates and by an optical microscope (Leica), the cultures were judged axenic. Axenic isolates were stored at −80°C in BG11 medium supplemented with 5% DMSO (vol/vol).

*Synechocystis* sp. PCC 6803 and *Synechococcus elongatus* PCC 7942 were used when needed as a standard cyanobacterial strain. For UV-C studies, *E. coli* DH10B was used as a reference. For conjugation experiments, *E. coli* HB101 was used. Cells were grown at 37°C in LB medium, supplemented with 50 μg mL^−1^ Km and 34 μg mL^−1^ Cm. Plasmids and strains related to this work are listed in Table 1.

**Table 1:** Plasmids and strains used in this study.

Strain/Plasmid	Description	References/Source
Strain		
*E. coli* HB101	*E. coli* conjugal strain	Lab. collection
*E. coli* DH10B	*E. coli* for UV-C experiments	Lab. collection
*Synechocystis* sp. PCC 6803	*Synechocystis* 6803, Wild type	Dr. Luis López-Maury (IBVF-CSIC)
*Synechococcus elongatus* PCC 7942	*Synechococcus* 7942, Wild type	Juan Nogales (CNB-CSIC)
Plasmid		
pSEVA 321	Replicative plasmid/shuttle vector, Cm^R^, ori RK2	Silva-Rocha et al. (2013)
pSEVA 331	Replicative plasmid/shuttle vector, Cm^R^, ori pBBR1	Silva-Rocha et al. (2013)
pSEVA 341	Replicative plasmid/shuttle vector, Cm^R^, ori pRO1600/ColE1	Silva-Rocha et al. (2013)
pSEVA 351	Replicative plasmid/shuttle vector, Cm^R^, ori RSF1010	Silva-Rocha et al. (2013)
pSEVA 351-Cpf1	Replicative plasmid/shuttle vector, Cm^R^, ori RSF1010 with Cpf1 cassette	Baldanta et al. (2022)
pRK2013	Conjugative plasmid (Km^R^), colE1 oriV replaces RK2 oriV, RK2 oriT, provide *tra* genes and nicking function from RK2	Elhai and Wolk (1988)

### 2.2. Molecular identification and phylogenetic analysis

Five milliliters of each isolate were harvested by centrifugation and washed 3 times to reduce the bacteria and extracellular substances present. Then, a rapid DNA extraction with chloroform was made for each cyanobacterial isolate. Single colonies growing on solid media were removed with a sterile plastic tip and resuspended in 100 μL of sterile deionized water. The same volume of chloroform (100 μL) was added to the suspension, vortex for 15 s, and then the mixture was centrifuged at 16.000 *g* for 15 min at room temperature. 50 μL of the upper, aqueous phase, were transferred to a new tube and used as DNA template for the different PCR applications. The rest of the mixtures were stored at 4°C until use. When bacteria were grown in liquid media, 200 μL culture were centrifugated to obtain a pellet that was washed with sterile deionized water, resuspended in 100 μL water and finally DNA was extracted as previously mentioned.

Polymerase chain reaction (PCR) analysis was carried out using primers for the amplification of the 16S rRNA gene or phycocyanin operon (Supplementary Table S2). PCR amplicons were cleaned with an NZYGelpure kit (Nzytech) according to the manufacturer’s instructions and then directly sequenced at Eurofins Genomics. All the sequence data obtained were analyzed using DNASTAR (Lasergene) programs. Alignments of nucleotide sequences were performed with BLAST.^1^

16 rRNA gene sequences from the identified *Chroococcidiopsis* isolates and the 4–5 most similar sequences were used for phylogenetic analysis. Multiple sequence alignments were performed using MUSCLE algoritm in MEGA-X. *Gloeocapsopsis dulcis* strain AAB1 (NR_172677.2) was added as an out-group. A neighbor-joining phylogenetic tree was built with the aligned sequences based on the K2 + G + I model with a bootstrap analysis involving 1,000 resampling trees using MEGA-X package.

### 2.3. Characterization of growth traits

Axenic isolates of *Chroococcidiopsis* sp. B11, B13, B14, and B15 were inoculated into 20 mL of BG11 medium in 100 mL Erlenmeyer flasks to an initial optical density at 750 nm (OD_750nm_) of 0.05 and grown at different salinity conditions (*via* NaCl concentration), pH, or nitrogen sources. For temperature experiment tests, 100 mL Erlenmeyer flasks with an initial OD_750nm_ of 0.20 were used. As a control, isolates grown under routine conditions (BG11 pH 7.5, 30°C, 150 rpm, and continuous light 100 μmol photon m^−2^ s^−1^) were used. Cell growth was monitored by measuring the OD_750nm_ for a 10-day period. In all the growth experiments, three biological replicates were performed. For *Chroococcidiopsis* sp. B13 measurements, as it grows in cellular aggregates, 1 mL of culture was first sonicated until aggregates were separated, and then cell integrity was checked by microscopy. To define the relationship between cell density per unit OD at 750 nm wavelength, a hemocytometer was used to count the cells.

To assess growth at different salt concentrations, BG11 medium was prepared containing 0.1, 0.25, 0.5, or 1 M of NaCl. The influence of pH on cyanobacterial growth was explored in BG11 buffered to pH 4, 9, and 11 with 10 mM Tris adjusted to each pH. The temperature effect on growth was evaluated at 4, 40, and 50°C, using 30°C as control. To examine the isolates for growth on different nitrogen sources, BG11 was modified by replacing the 16 mM of NaNO_3_ with 16 mM of NH_4_Cl or urea. Tolerance to urea was determined by adding this compound to final concentrations of 8 and 16 mM to BG11.

All the strains were inoculated at 0.05 OD_750nm_ from a 7–10-days old culture and OD measurements were taken every day for 10 days. To calculate growth rates and doubling time, we plotted logOD versus time (hours), and using *r* = (log [OD2/OD1]) / (T2–T1) in the linear portion. Doubling time corresponds to log2/r. The beginning of the growth phase was considered when the growth of the cyanobacteria was appreciable. One-way Anova with post-hoc Tukey HSD, with Scheffé, Bonferroni and Holm multiple comparison results was calculated for each strain on Astatsa.com (Vasavada, 2016).

### 2.4. Heterotrophic growth

To assess the heterotrophic growth of axenic isolates, BG11 agar plates were prepared at final concentrations of 10 mM with different carbon sources: glucose, sucrose, lactose, arabinose, maltose, fructose, galactose, mannose, and glycerol. The tests were performed with spots of 10 μL at OD_750nm_ = 1 onto BG11 plates to reduce the possibility of contamination. Plates were incubated at 30°C in darkness for 30 days. Furthermore, the photosynthesis inhibitor DCMU [3-(3,4-dichlorophenyl)-1,1-dimethylurea] was added for a final concentration of 10 μM to make sure that the observed growth was heterotrophic. Isolates were checked to ensure that they were free of contaminant bacteria before the experiments.

### 2.5. Carbohydrate quantification

To determine the total carbohydrate content in the different cultures, phenol-sulfuric acid method was applied as described (Dubois et al., 1956), modified to a microplate format (Masuko et al., 2005). 5 mL samples of 1-month-old culture of each *Chroococcidiopsis*. isolate, grown without shaking, were used for carbohydrate quantification. This procedure was carried out in the BIOPEN-IMDEA Energy laboratories.

### 2.6. Biofilm assay

Biofilm formation by *Chroococcidiopsis* sp. Helios was assayed using crystal violet staining described previously with slight modifications (Allen et al., 2019). Briefly, three milliliters of 2-to 3-week-old *Chroococcidiopsis* sp. B11, B13, B14, and B15 were cultured in M6 plates at an initial OD_750nm_ of 0.2, and maintained at 30°C in continuous light (100 μmol photon m^−2^ s^−1^) without shaking for 3 days. As a control, BG11 media without cells were used. After removing the non-adherent cells by aspiration of the medium, cells were washed twice with 2 mL of PBS, and then 0.3% crystal violet in PBS (wt/vol) was added for 10 min to stain the adherent cells. After removing the stain, cells were washed with 2 mL of PBS at least three times. The cells were then suspended in 10 mL of 95% ethanol (vol/vol) for 30 min, and the Abs_588nm_ was measured. Measurements correspond to cells adhered to the glass. All assays were replicated at least three times.

### 2.7. Desiccation-tolerance test

Cyanobacterial isolates were grown on 9–10 mL of BG11 agar plates (6 cm diameter) under light (60–80 μmol photon m^−2^ s^−1^) at 30°C for 2 weeks with a 30–35% relative humidity in the air. Then, plates were left to be air-dried at under routine growth conditions by removing the parafilm from the Petri dishes. After about 15 days, dried cultures were stored in the laboratory bench room temperature for 3 months, 7 months, and 1 year (on benchtop, RT, 10 μmol photon m^−2^ s^−1^ during the day). For the 1-year dried samples, some samples were maintained in parallel under routine growth conditions. For rehydration, the dried samples were soaked with 1 mL of sterile water for 15 min at room light, streaked on BG11 plates, and incubated under 60–80 μmol photon m^−2^ s^−1^ and 30°C. Results were observed after 2–3 weeks. As a negative control for desiccation tolerance, *Synechocystis* sp. PCC 6803 was used.

### 2.8. UV-C irradiation test

To assess the viability of the isolates to radiation, axenic cultures of cyanobacteria were exposed to different doses of UV-C. Cultures in the late exponential phase of growth were prepared at an OD_750nm_ of 0.5 in BG11 medium for cyanobacteria, OD_600_ of 0.5 in PBS, for bacteria. We used *E. coli* and *Synechococcus elongatus* PCC 7942 as model bacteria for low and moderated UV-C resistance, respectively. Samples without UV-C irradiation were also set as controls. Aliquots of 2 mL were placed in uncovered 6-well cell culture plates (3 cm in diameter) and irradiated with a 460 mW m^−2^ UV-C lamp. The irradiation assays were performed in room-light conditions and the doses were adjusted to 250, 500, 750, and 1,000 J m^−2^ varying exposure times. The absorbance of BG11 medium at 254 nm was reported to be 0.09 (Tao et al., 2010). Survival after UV-C exposure was tested by colony-forming ability. After irradiation, 1 mL of each sample was collected; then, decimal dilutions were prepared and plated onto agarized BG11 for cyanobacteria and LB for *E. coli*. Cyanobacterial and bacterial colonies were scored after 2 weeks and 1–2 days of incubation, respectively. The loss of viability was determined by expressing the cell viability as a ratio of the number of viable cells in UV-C-exposed samples to the control (non-exposed) cells calculated as a percentage.

### 2.9. Antibiotic sensitivity evaluation

The antibiotic sensitivity of the axenic cultures was evaluated against a set of several antibiotics. Spots of each isolated were plated (10 μL) in BG11 agar with different anbiotics and incubated at 30°C for 7 days. Several concentrations for each antibiotic were tested: 7.5–75 μg mL^−1^ chloramphenicol (Cm), 20–100 μg mL^−1^ kanamycin (Km), 25–250 μg mL^−1^ neomycin (Nm), 2–20 μg mL^−1^ streptomycin (Sm), 2–20 μg mL^−1^ spectinomycin (Spt), 2–20 μg mL^−1^ gentamicin (Gm), 20–200 μg mL^−1^ erythromycin (Em), and 7.5–75 μg mL^−1^ nalidixic acid.

### 2.10. Gene transfer by triparental mating

A set of cargo-mobilizable plasmids (Table 1) were selected for transfer to the axenic cyanobacterial isolated by spot triparental mating, following published protocols (Elhai and Wolk, 1988). Briefly, conjugation of the different plasmids into all isolates was performed by mixing 0.75 mL overnight cultures of HB101 pRK2013 (conjugative strain) and 1 mL of HB101 cargo plasmid and concentrating the cells in 60 μL. The *E. coli* mix was incubated for 1 h at 37°C without shaking. On the other hand, cultures 2–3 weeks old of *Chroococcidiopsis* were centrifuged and the OD_750nm_ was adjusted from 8 to 0.08 in order to test different ratios of cyanobacteria:bacteria for the mating process. After that, 10 μL was mixed with 10 μL of the cyanobacteria isolate. Mating was carried out on Immobilon-NC filters resting on BG11 agar supplemented with 5% LB for 48 h under conditions used for maintenance growth. Then, filters were transferred to BG11 plates with the appropriate antibiotics. After incubation for 1–2 weeks, isolated transconjugant colonies were patched on fresh selective BG11 plates. The presence of replicative plasmids was confirmed by PCR using specific primers (Supplementary Table S2).

### 2.11. Construction and evaluation of a recombinant plasmid based on the pSEVA 351

To construct a pSEVA 351-based plasmid, the synthetic strong cyanobacterial promoter P_c223_ was used to constitutively express the *yfp* gene (Markley et al., 2015). The construct was chemically synthetized (Thermofisher) and amplified by PCR using Q5 High-Fidelity DNA Polymerase (New England Biolabs) according to the manufacturer’s protocol. The resulting PCR product was then digested with *Pml*I y *Pst*I and cloned into pSEVA351 digested with *Sma*I and *Pst*I. The developed plasmid, named pSEVA351-YFP, was transferred onto *Chroococcidiopsis* sp. Helios by triparental mating (described above). After plasmid presence confirmation, the transformants were grown on solid BG11 Cm10. After a week, cells were resuspended in 200 μL of liquid BG11 and the OD_750nm_ was measured. The fluorescence of the different cultures was quantified by transferring 100 μL adjusted to OD_750nm_ 0.1 in 96-well transparent bottom black plates and measured with a Varioskan reader, using 485/20 and 535/25 nm filters for excitation and emission, respectively. Relative fluorescence was normalized to OD_750nm_ (Fluorescence/OD_750nm_).

## 3. Results

### 3.1. Diversity of cyanobacteria isolated from the solar panel

Samples collected from solar panels in Valencia were initially plated on BG11, UTEX, MDM, and Castenholz-D medium. In parallel, two of these media were also used without a combined nitrogen source: MDM_0_ and BG11_0_. No colony could be observed in either of the media lacking a source of nitrogen, but a total of 39 viable colonies were observed in BG11, UTEX, and Castenholz-D. After 2 months of cultivation, only 15 of these viable colonies survived. Single colonies were picked up from each medium, diluted, and streaked again on fresh agar-containing plates. Eight of these isolates were filamentous and seven unicellular, with coccoid form. No fungal contaminations were observed at this step. To eliminate bacterial contamination, isolates with the best growth were re-streaked until no bacteria were detected, as described in MM section 2.1.

Twelve out of fifteen viable colonies were identified by 16S/18S rRNA gene sequence analysis, which revealed that both cyanobacteria (10 isolates) and green algae (two isolates) are present in a solar panel (Table 2). All the best BLAST hits listed in Table 2 are commonly identified in arid locations, where low water availability and high irradiance rates are characteristic (Supplementary Table S3). The 10 cyanobacterial isolates were classified into two orders, including Chroococcidiopsidales and Synechococcales, according to their 16S rRNA sequence (NCBI database). The members in Synechococcales were from various genera including *Leptolyngbya*, *Oculatella*, and *Myxacorys*. Moreover, the green algae *Diplosphaera* sp. and microalgae *Coelastrella* sp. (isolates B7 and D14, respectively) were also identified in the sample taken from the solar panel.

**Table 2:** BLAST results obtained by querying the 16S/18S rRNA gene of the different cyanobacteria from a solar panel with GenBank.

Isolate ID	Closest BLAST sequence	Identity %	Accession
B1	*Oculatella coburnii* WJT66-NPBG6A	99.73	KF761586.1
	*Oculatella mojaviensis* CMT-3BRIN-NPC87	99.73	KF761572.1
B2	*Oculatella coburnii* WJT66-NPBG6A	100	KF761586.1
	*Oculatella mojaviensis* CMT-3BRIN-NPC87	100	KF761572.1
B5	*Oculatella coburnii* WJT66-NPBG6A	100	KF761586.1
	*Oculatella ucrainica* KZ-5-4-1	90.89	MG652620.1
B6	*Oculatella coburnii* WJT66-NPBG6A	99.73	KF761586.1
	*Oculatella mojaviensis* CMT-3BRIN-NPC87	99.73	KF761572.1
B7	*Diplosphaera chodatii* strain SAG 49.86	98.85	MT078182.1
	*Diplosphaera chodatii* strain SAG 9.82	98.85	MT078181.1
	*Diplosphaera chodatii* strain SAG 2049	98.85	MT078180.1
B11	*Chroococcidiopsis* sp. CENA240	99.82	MN551907.1
	*Chroococcidiopsis* sp. CCNUC3	99.33	MN544283.1
B13	*Chroococcidiopsis* sp. CENA246	100.00	MN551908.1
	*Chroococcidiopsis* sp. CENA240	100.00	MN551907.1
	*Chroococcidiopsis* sp. CCNUC1	100.00	MN544281.1
B14	*Chroococcidiopsis* sp. CENA246	100.00	MN551908.1
	*Chroococcidiopsis* sp. CENA240	100.00	MN551907.1
	*Chroococcidiopsis* sp. CCNUC1	100.00	MN544281.1
B15	*Chroococcidiopsis* sp. YRS 4a	95.33	KF908844.1
	*Chroococcidiopsis* sp. SAG 2025	95.33	AM709635.1
B16	*Myxacorys californica* WJT24-NPBG12	99.1	MF996894.1
	*Myxacorys californica* FI6-MK20	99.1	KJ939075.1
	*Myxacorys californica* CMT-1FSIN-NPC23	99.1	KJ939073.1
D14	*Coelastrella* sp. QW-2019b	99.0	MH176108.1
UTEX1	*Leptolyngbya* sp. RV74	98.81	KP681566.1

**Figure 1:**
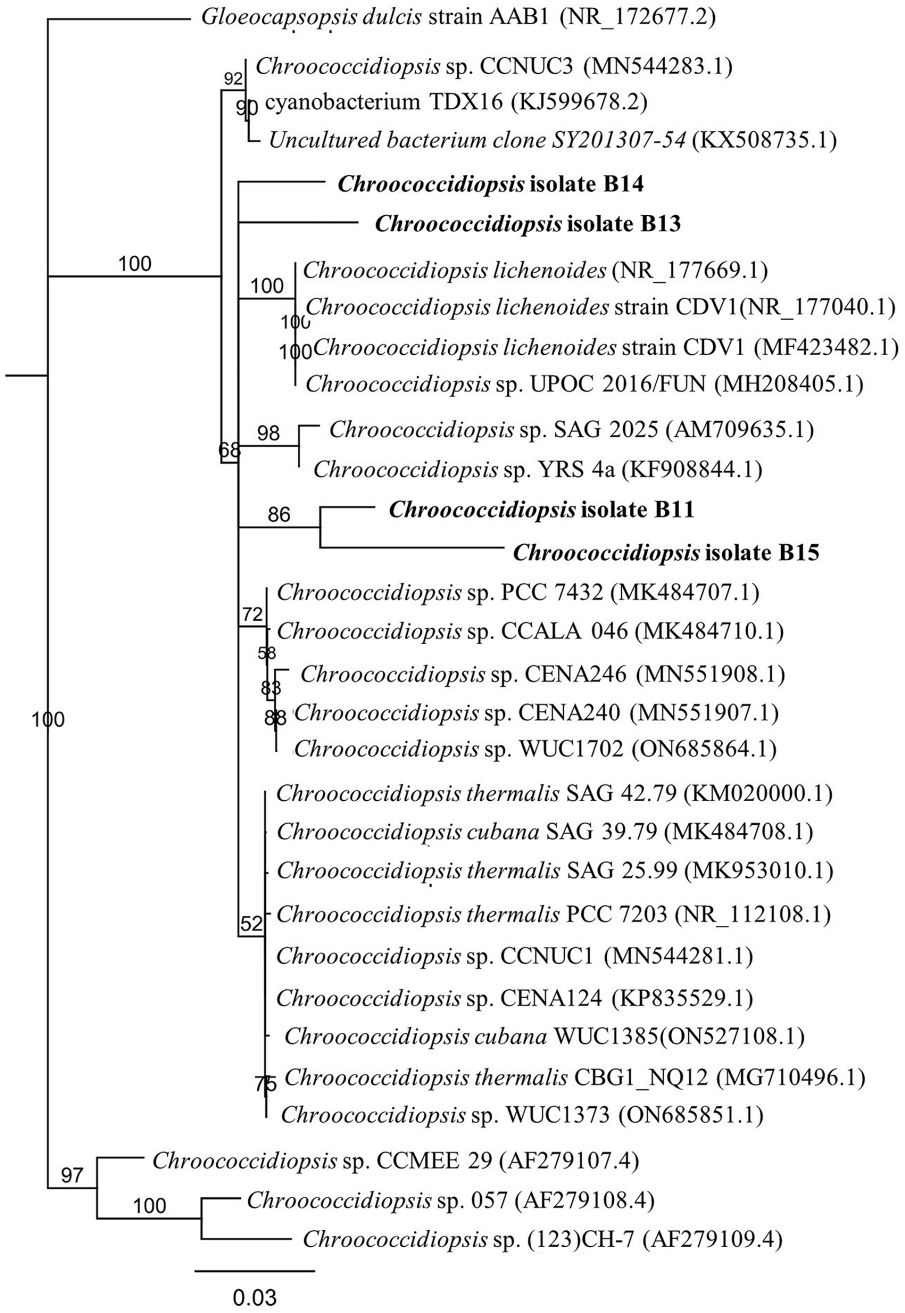
Phylogenetic tree inferred from 16S rRNA gene sequences from the *Chroococcidiopsis* isolates from the solar panel samples. The tree was constructed using the neighbor-joining method in MEGA-X. The length of the scale bar indicates 0.2 substitutions per site. The percentages of bootstrap support of branches (>50%) are indicated at each node. The isolates from the solar panel are shown in bold.

Of all the cyanobacterial isolates, only *Chroococcidiopsis* isolates were unicellular or packet forming, facilitating their handling in the laboratory. The *Chroococcidiopsis* isolates also had a faster growth rate than other isolates. Taking this into account, we chose the cyanobacterial isolates *Chroococcidiopsis* sp. B11, B13, B14, and B15 for further characterization (hereafter referred to as Helios isolates or *Chroococcidiopsis* sp. Helios). The phylogenetic analysis with the *Chroococcidiopsis* isolates and the 4–5 most similar sequences obtained in BLAST (NCBI database), shows that B13 and B14 are clustering together while B11 and B15 lie in a different branch (Figure 1).

### 3.2. Growth characterization of Helios isolates

**Figure 2:**
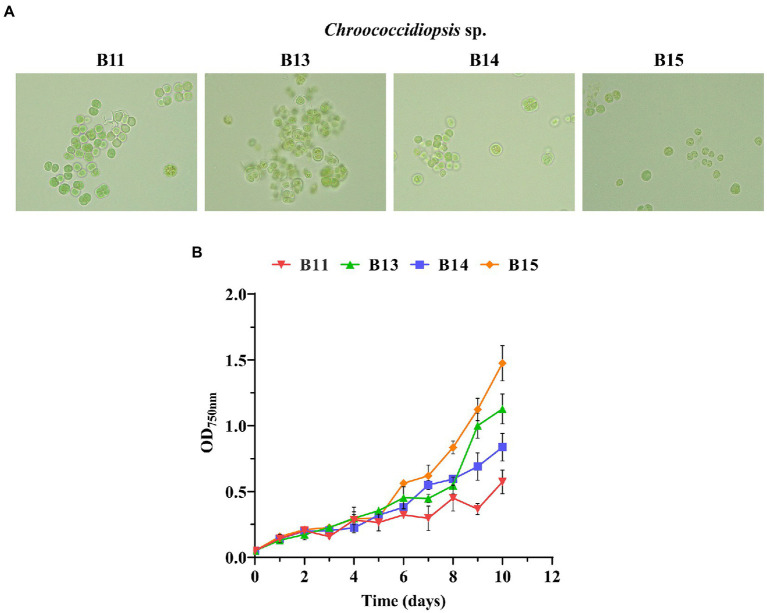
Growth curves of *Chroococcidiopsis* sp. Helios isolates. **(A)** Bright-field photomicrographs of the chosen isolates taken at the same magnification (100X). **(B)** Growth curves of some of the isolates. The graph shows the average OD_750nm_ of three biological replicates together with the standard deviation.

To characterize the growth traits of the chosen isolates, the optical density at 750 nm (OD_750nm_) of *Chroococcidiopsis* sp. B11, B13, B14, and B15 was measured over 10 days in a range of salinity, pH, temperature, carbon, and nitrogen sources. The isolates displayed different growth rates in the conditions assayed (routine maintenance) and consisted of BG11 with pH 7.5, 30°C, 150 rpm, and continuous light (100 μmol photon m^−2^ s^−1^) (Figure 2 and Supplementary Table S4). The relationship between the cell concentration (cell density) and the corresponding optical density at 750 nm (OD_750nm_) for the different cyanobacteria was also obtained (Supplementary Table S4). *Chroococcidiopsis* sp. B15 was the fastest isolate with a doubling time of under 3 days. The other isolates had similar growth rates, being B11the lowest one with a doubling time of 6.6 days. All Helios isolates were pale green, rounded, and tended to be grouped mainly in pairs or tetrads. *Chroococcidiopsis* sp. B13 had the highest tendency to be aggregated, gathering many tetrads during its growth. It was necessary to sonicate the culture before measurement.

**Figure 3:**
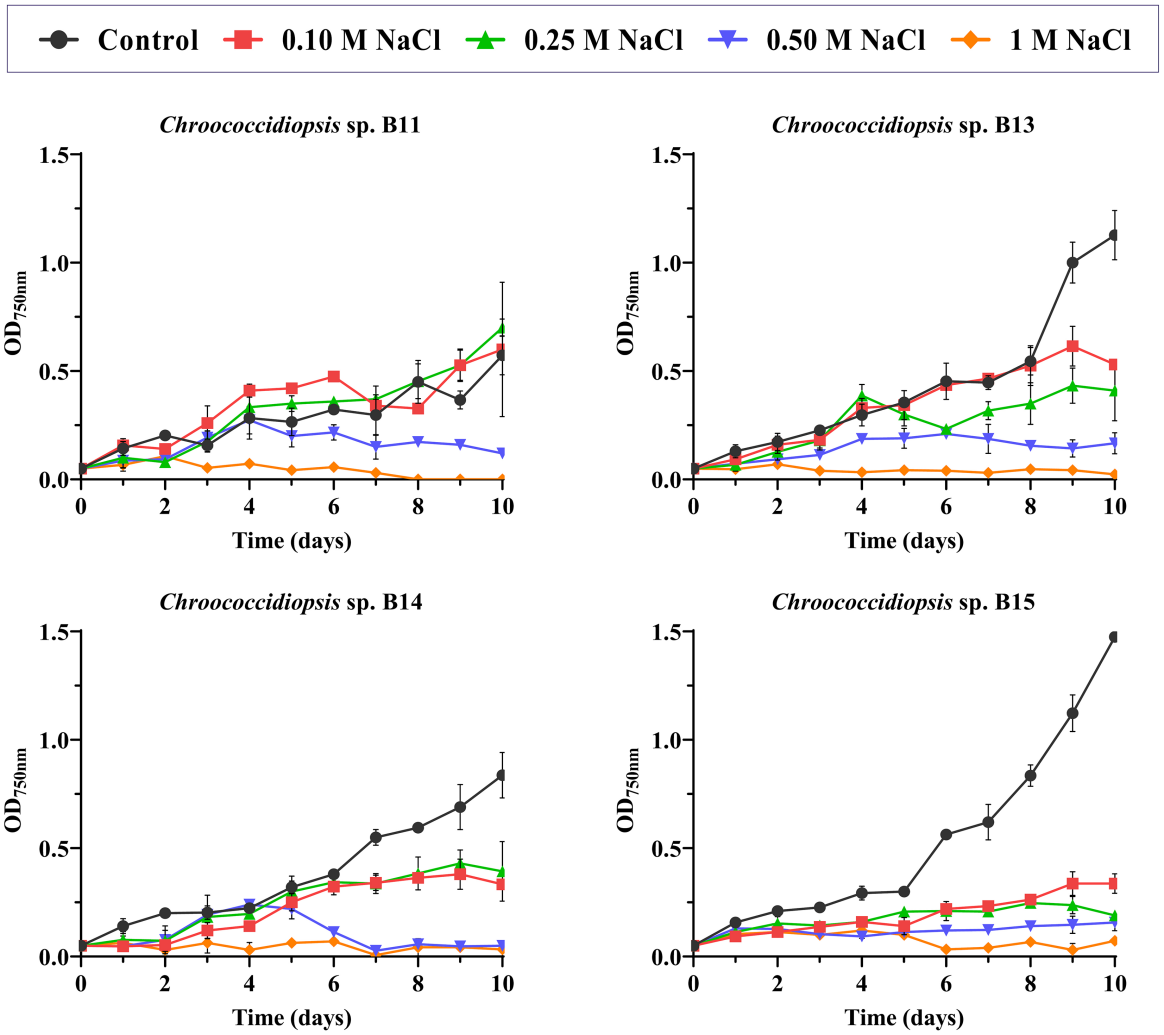
Effect of NaCl concentration in Helios isolates growth. As a control, growth in BG11 medium was used. The graph shows the average of three biological replicates together with the standard deviation. The graphs show the average OD_750nm_ of three biological replicates together with the standard deviation.

Regarding salt stress, not all the isolates showed the same pattern of response to increasing NaCl concentration (Figure 3 and Supplementary Tables S5–S13). Statistical analysis showed that *Chroococcidiopsis* sp. B11 growth was clearly improved by the addition of 0.1 M NaCl, since the doubling time was shorter (from 6.7 to 2.8 days) and the maximal absorbance was similar to the control (BG11 medium) (Figure 3 and Supplementary Tables S5, S6). On the other hand, B15 growth was sensitive to NaCl, with doubling time values longer (from 2.4 days of the control to 8.3 days at 0.5 M NaCl) and maximum absorbance values lower than the control in all the concentrations of NaCl tested (Figure 3 and Supplementary Tables S11, S12). B13 was also affected by NaCl presence, mainly by decreasing the maximum absorbance values with the NaCl concentration (Figure 3 and Supplementary Tables S7, S8). Isolate B14 growth was affected mostly at 0.5 M NaCl (Figure 3 and Supplementary Tables S9, S10). No growth was observed at 1 M of NaCl in all *Chroococcidiopsis* isolates.

The effect of pH on growth was also evaluated and the best pH for growth was the control with 7.5, for all the isolates (Supplementary Figure S1). *Chroococcidiopsis* sp. B13 was able to grow both at pH 7.5 (control) and 9 while the statistical analysis showed that there was not significant differences between these two conditions (Supplementary Table S7). For *Chroococcidiopsis* sp. B11, B14, and B15, no growth was detected when the pH was changed, although some cultures did not bleach.

Not only pH but also temperature greatly affected the growth of *Chroococcidiopsis* sp. (Supplementary Figure S2). None of the isolates were able to grow at 50°C in liquid medium, and the cultures were bleached at the second day of the experiment. According to the statistical analysis the growth was significantly impaired at 4 and 40°C (Supplementary Tables S6, S8, S10, S12). However, at 40°C, after 10 days, the cultures remained green and viable.

**Figure 4:**
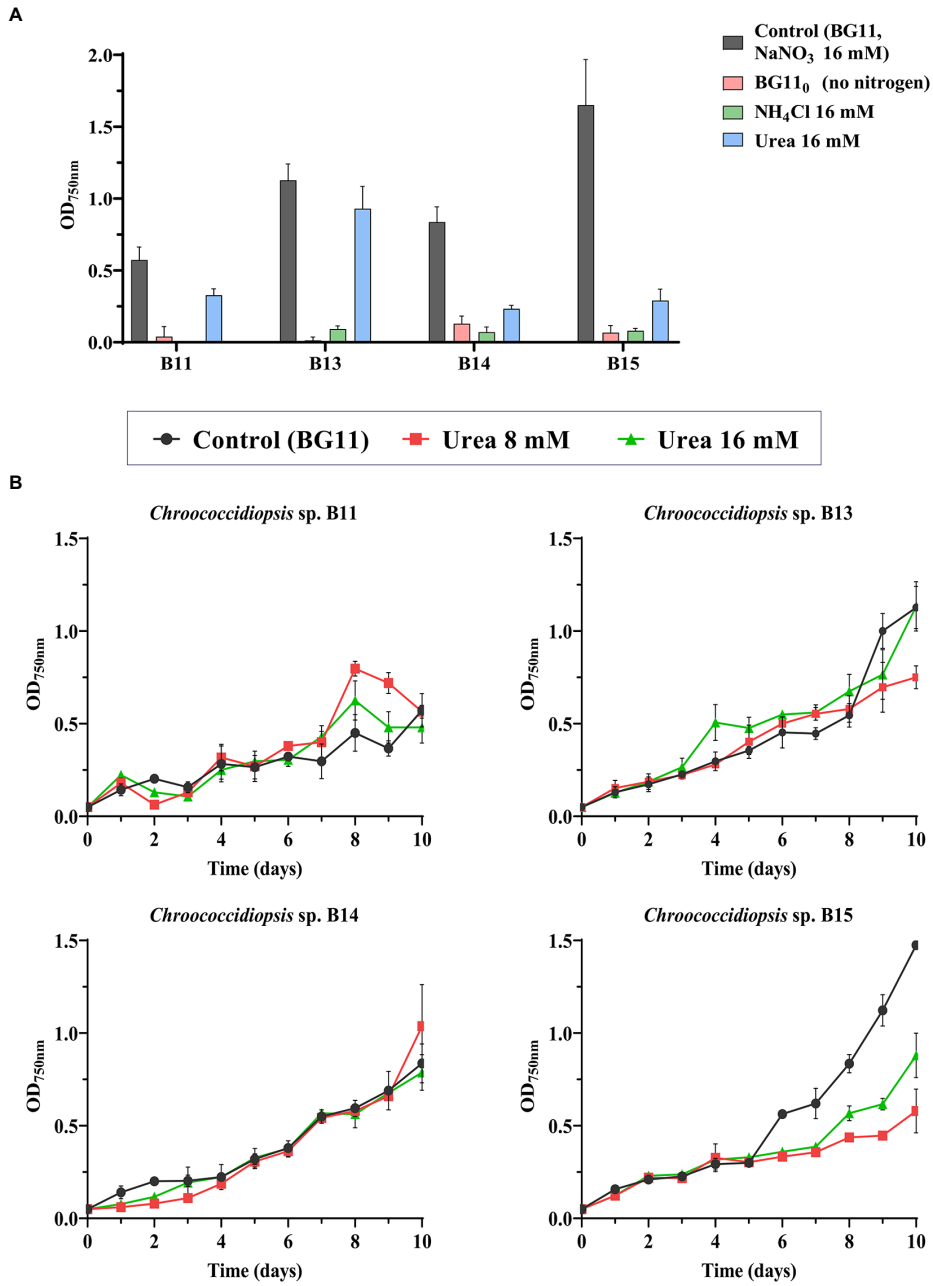
Effect of nitrogen source on the growth of *Chroococcidiopsis* sp. **(A)** OD_750nm_ after 10 days of growth using different nitrogen sources. As control, BG11 was used (NaNO_3_ 16 mM). In NH_4_Cl and urea treatments, NaNO_3_ in BG11 was substituted for urea or NH_4_Cl (16 mM). Alternatively, no nitrogen was added (BG11_0_). **(B)** Tolerance to urea in the different *Chroococcidiopsis* isolates. Urea at 8 and 16 mM was added to BG11 complete medium (containing NaNO_3_ 16 mM). The graphs show the average OD_750nm_ of three biological replicates together with the standard deviation.

Although we initially used nitrate as a source of nitrogen to grow the different *Chroococcidiopsis* isolates, the effect of its absence or the use of other nitrogen sources for the cell growth was assessed in liquid cultures. All nitrogen sources were prepared at 16 mM, the same concentration as NaNO_3_ used in BG11. Figure 4A shows the final OD_750nm_ after 10 days using alternative nitrogen sources, such as urea or ammonium. None of the isolates were able to grow without any nitrogen source, which may be because they lacked the ability to fix atmospheric nitrogen in the conditions tested. Growth in the presence of ammonium was also closer to the result observed in BG11_0_. However, even though all the isolates achieved the maximal absorbance with nitrate, it seems that urea could also be used as a nitrogen source for most of the isolates. In terms of the maximal absorbance only B13 isolate did not presented a significant difference to NaNO_3_ under urea concentrations of 16 mM (Figure 4A and Supplementary Table S8). The rest of assayed isolates were affected when growing in 8 mM urea and ammonium (Figure 4A and Supplementary Tables S6, S10, S12).

Considering that urea can be abundant in wastewater, in order to explore the possibility to use these strains in bioremediation, we tested the growth of the different isolates in BG11 with different concentrations of urea (Figure 4B). According to the statistical analysis, the doubling time (9.7 days) was not affected in strains B13 and B14 growing in the presence of urea (Supplementary Tables S7, S9) or even in the case of B11, a shorter doubling time was observed (from the control at 6.7 days to 2.1 days at 16 mM urea), indicating that the presence of urea, probably as an extra source of nitrogen, seems to improve this grow (Supplementary Table S5). By the contrary, the maximal absorbance of isolate B15 decreased in all urea treatments (8 and 16 mM) (Figure 4B and Supplementary Table S12). These results show that even though urea cannot replace NaNO_3_ as a nitrogen source, some isolates tolerate it.

Finally, *Chroococcidiopsis* isolates were tested for heterotrophic growth. We evaluated growth using glycerol and eight different sugars at 10 mM in BG11 agarized medium in complete darkness, and with the photosynthesis inhibitor DCMU at 10 μM final concentration for 30 days. *Chroococcidiopsis* sp. B11, B13, B14, and B15 were able to grow heterotrophically in the presence of the disaccharides sucrose and maltose but not lactose (Table 3 and Supplementary Figure S3). Regarding monosaccharides, all the isolates tested seem to grow slowly in glucose, while in fructose, only B13 and B14 isolates showed slight growth. Conversely, no growth was detected in mannose, galactose, arabinose, and glycerol for all the isolates tested.

**Table 3:** Heterotrophic growth of *Chroococcidiopsis* isolates.

		*Chrooccocidipsis* sp.			
Carbon source		B11	B13	B14	B15
Monosaccharide	Glucose	+/−−	+/−−	+/−−	+/−−
	Fructose	−	+/−−	+/−−	−
	Mannose	−	−	−	−
	Galactose	−	−	−	−
	Arabinose	−	−	−	−
Disaccharide	Sucrose	+	+	+	+
	Maltose	+	+	+	+
	Lactose	−	−	−	−
Alcohol	Glycerol	−	−	−	−

+, robust growth; +/−−, small colonies; −, no growth.

### 3.3. Polysaccharide composition and biofilm assay

**Figure 5:**
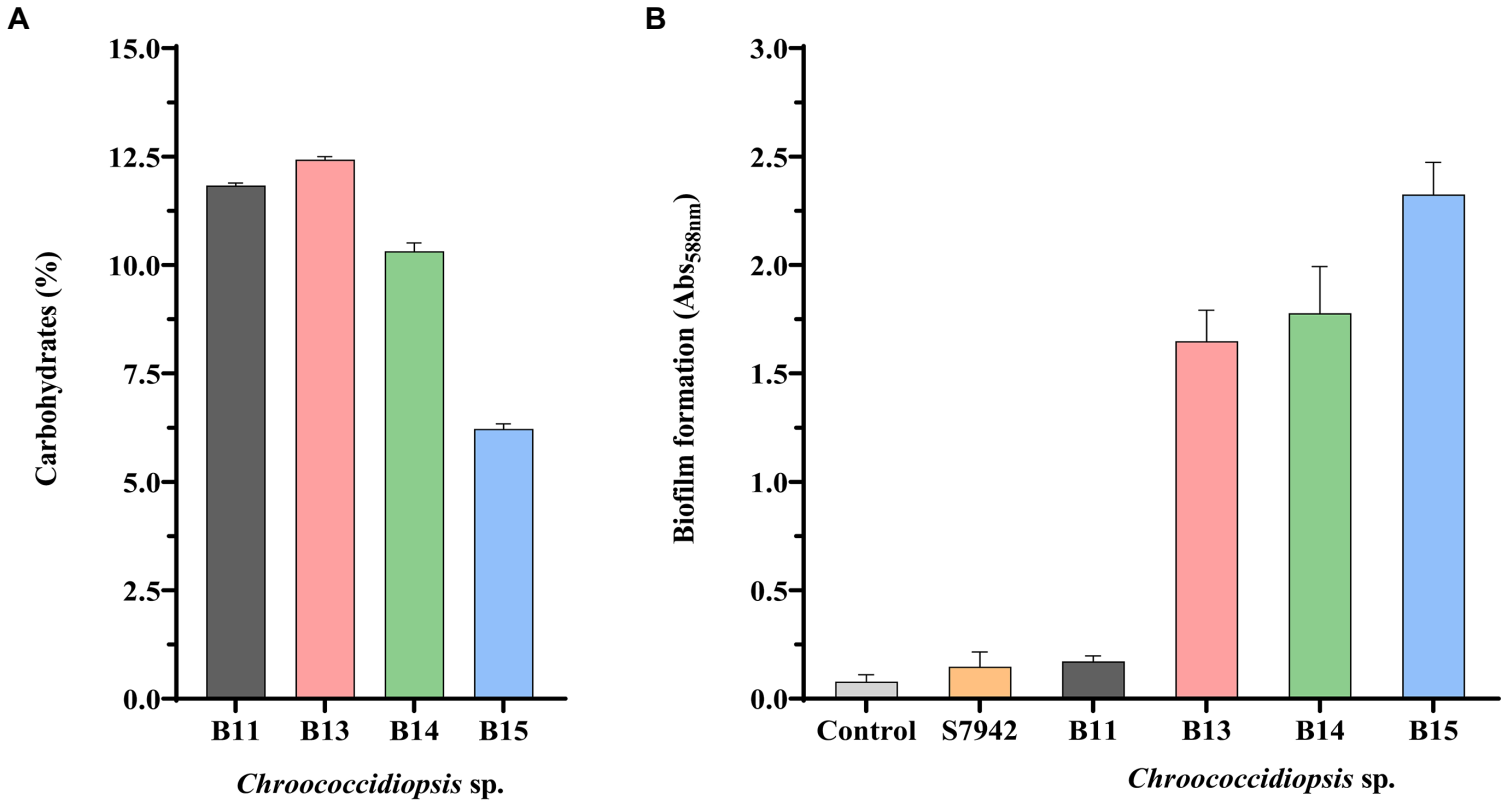
Carbohydrate content **(A)** and biofilm formation **(B)** in *Chroococcidiopsis* isolates. The bar charts show: **(A)** total carbohydrates content per cell (dried weight) (%), and **(B)** crystal violet staining quantification by measuring absorbance (Abs_588 nm_). S7942 is *Synechococcus elongatus* PCC 7942. Wells without cells were used as a control. The graphs show the average of three biological replicates together with the standard deviation.

To further evaluate differences among isolates, the percentage of carbohydrate content was determined in the four *Chroococcidiopsis* isolates (Figure 5A). Isolate B15 with 6.22% had almost half the carbohydrate content of the isolates B11, B13, and B14 (10.32–12.43%). On the other hand, we measured the ability of *Chroococcidiopsis* isolates to form biofilms (Figure 5B). Here we found that there are some differences among the isolates (Figure 5B). The only isolate that was not able to form biofilms was *Chrooccocidiopsis* sp. B11, which gave values similar to the control and *Synechococcus* 7942. The other isolates were able to form biofilms after 3 days without shaking, which is usual in extremophile bacteria (Figure 5B).

### 3.4. Effect of desiccation and UV-C exposure in *Chroococcidiopsis* isolates

**Figure 6:**
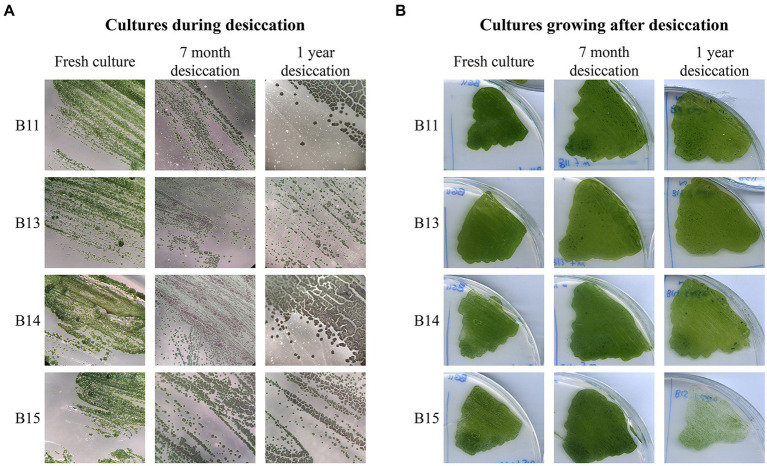
Long-term desiccation tolerance in *Chroococcidiopsis* isolates. **(A)** *Chroococcidiopsis* sp. Helios colonies throughout the desiccation process. Fresh cultures were included as a control. **(B)** Growth observed after 2 weeks on BG11 post rewetting for 7-month and 1-year desiccated culture; a freshly streaked isolate was included as a control.

Considering that the solar panel could be subjected to periodic absences of water, we investigated the desiccation-tolerance of *Chroococcidiopsis* isolates. Cultures were plated and subjected to dehydration for 3 months, 7 months, and 1 year, and then rewetted and cultured on solid BG11. Fresh cultures were used as non-desiccated controls. As shown in Figure 6, in terms of ability to form colonies and grow, the rewetted isolates differed little from the non-dried cultivated forms, indicating that all the isolates were drought-resistant. The same result was observed for almost all the isolates from the solar panel (Supplementary Figure S4). *Synechocystis* sp. PCC 6803, which was used as a non-resistant strain, was unable to grow after 3 months, the minimum time tested (Supplementary Figure S5). On the other hand, the effect of light was negative for dried cultures, as the plates kept in the conditions used for growth maintenance showed blue colonies and did not grow after rewetting (Supplementary Figure S5).

**Figure 7:**
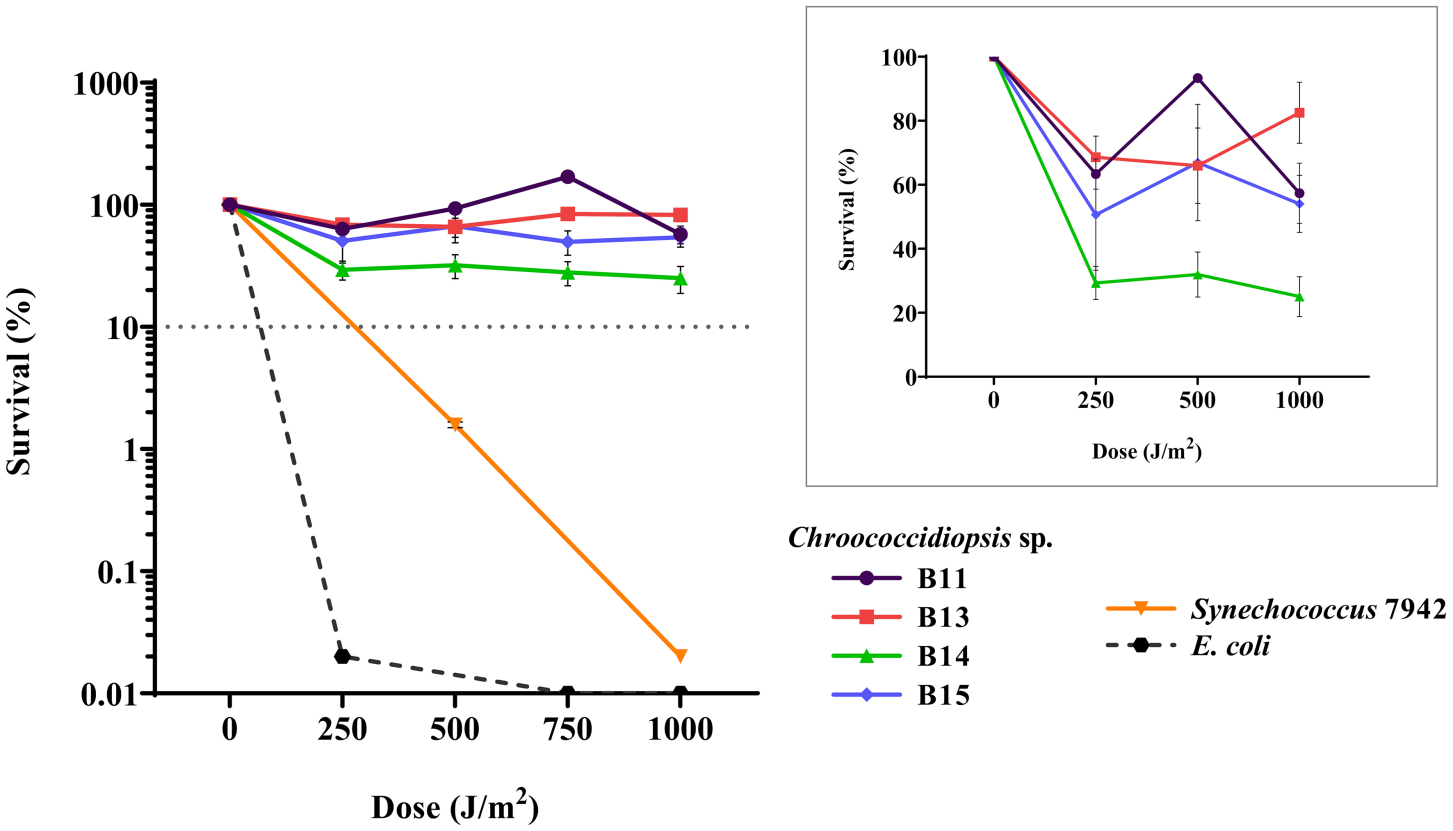
Viability of cyanobacterial cultures after exposure to UV-C radiation. The graph shows the averages of three biological replicates together with the standard deviation. The smaller graph shows the survival percentage just for *Chroococcodiopsis* sp. Helios.

Since the genus *Chroococcidiopsis* has been already proposed as an ideal candidate for astrobiological applications by several authors (Verseux et al., 2016; Billi et al., 2019; Bothe, 2019), we wanted to present a preliminary approach to UV-C irradiance resistance. Along with the desiccation tolerance, the resistance of Helios isolates to UV-C irradiance was tested. Different doses were used: 250, 500, 750, and 1,000 J m^−2^. Survival rates for all the isolates were similar (Figure 7) at above 50%, except for *Chroococcidiopsis* sp. B14, whose viability dropped below 30%. For comparison, the survival rate of *E. coli*, used as a UV-C-sensitive control, was around 0.02% at the first dose of UV-C exposure. In contrast, *Synechococcus* 7942, employed as medium UV-C-resistant control, also survived more than *E. coli* but far less than *Chroococcidiopsis* sp. Helios.

### 3.5. Conjugal transfer by triparental mating

In order to have a panel of antibiotics that could be used as selective markers in genetic manipulations, the antibiotic sensitivity of *Chroococcidiopsis* isolates was evaluated for eight antibiotics in BG11 agar media (Supplementary Table S13). All the isolates were sensitive to chloramphenicol, streptomycin, erythromycin, and neomycin, even at the lowest concentrations checked (25 μg mL^−1^), but they were resistant to kanamycin, spectinomycin, gentamicin, and nalidixic acid.

**Figure 8:**
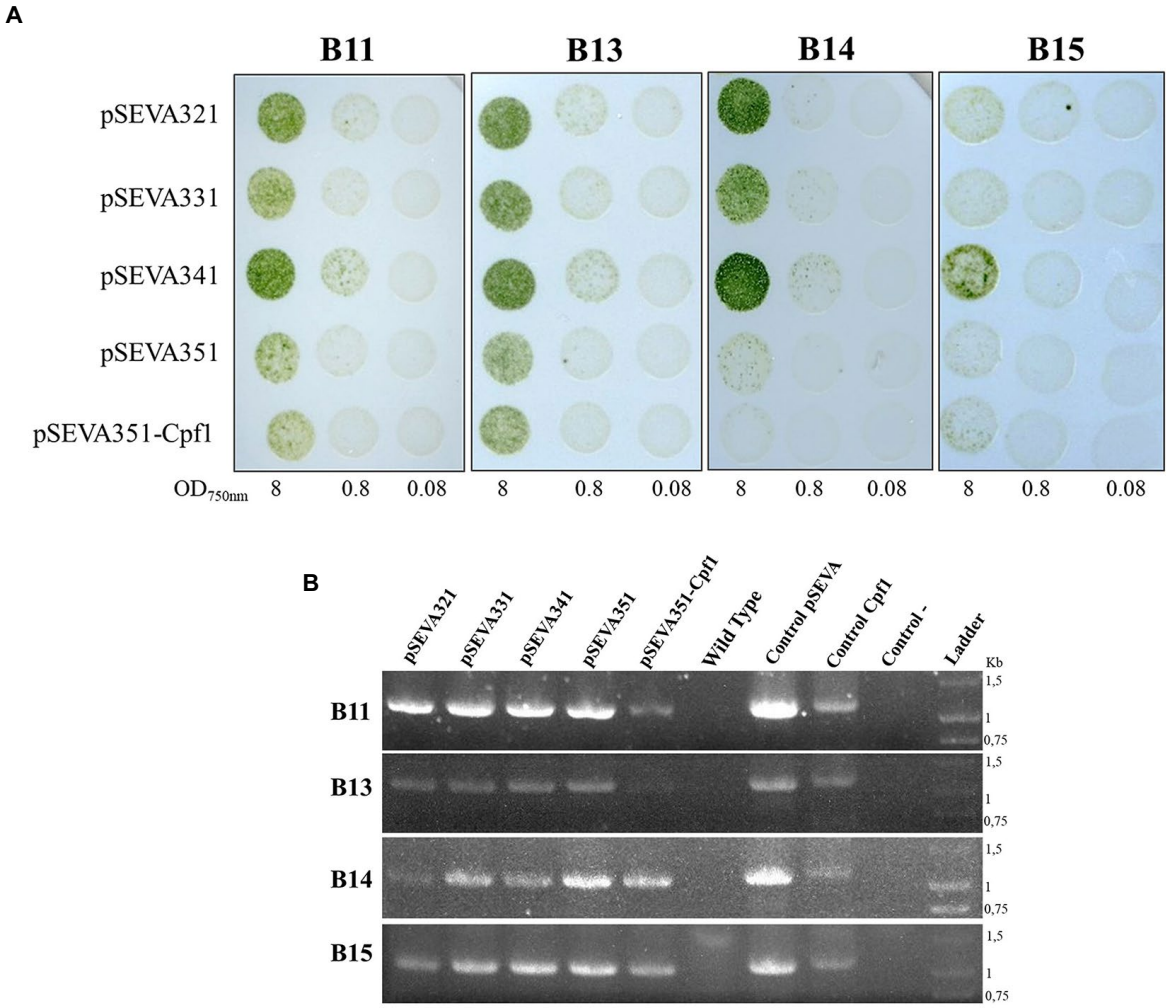
Triparental mating in *Chroococcidiopsis* isolates. **(A)** Result of the spot triparental mating for B11, B13, B14, and B15 isolates. Different OD_750nm_ were tested. **(B)** PCR confirmation of positive transformants of 7-day culture. Specific primers, amplifying chloramphenicol resistance gene in different plasmids, were used for verification (Supplementary Table S2): primers used for SEVA vectors (Oligo8/R24) yielded a 1 kb fragment; primers used for pSEVA with the CRISPR-Cas machinery (Oligo8/R24rv) yielded a 1.3 kb fragment. As positive controls, plasmid pSEVA351 (Control pSEVA) and pSEVA351-Cpf1 (Control Cpf1) were used; wild-type DNA of each isolate and water were used as negative controls.

To determine if Helios isolates could be transformed after conjugation with *E. coli* donor cells, a technique that has been used to introduce DNA into a wide variety of cyanobacteria, we performed triparental mating using pRK2013 as a conjugative plasmid, and different cargo plasmids (see Table 1). The chosen plasmids contain origins RSF1010, RK2, pBBR1, and pRO1600/ColE. Additionally, the RSF1010-derived plasmid, which included the CRISPR-Cas12(Cpf1) machinery, was tried. After conjugation, transconjugant colonies of B11, B13, B14, and B15 became apparent on selective mating plates after 1 week (Figure 8A). These isolates showed robust growth after being transferred to fresh selective BG11 Cm (10 μg mL^−1^) plates and the presence of the plasmid in the transformed isolates was successfully confirmed by PCR (Figure 8B). Thereafter, these results demonstrate that broad host range plasmid vectors based on RSF1010, RK2, pBBR1, and pRO1600/ColE origins can be efficiently transferred to all the Helios isolates (Figure 8A).

**Figure 9:**
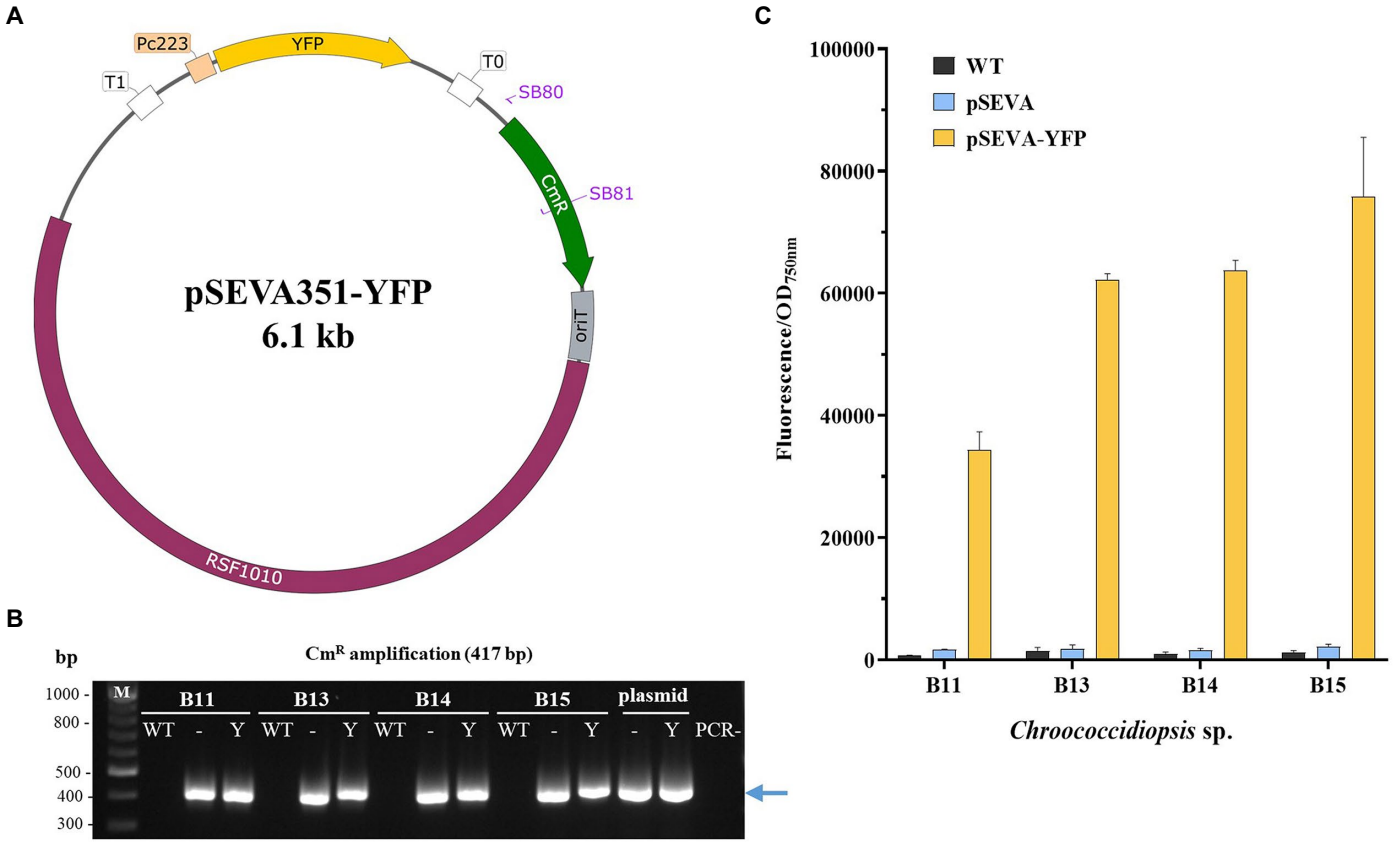
Construction and evaluation of the pSEVA351-YFP as an expression vector in *Chroococcidiopsis* sp. Helios. **(A)** Map of the pSEVA351 carrying the YFP reporter under the promoter P_c223_ (pSEVA351-YFP). **(B)** PCR confirmation of positive transformants of 7-day cultures. Specific primers SB80/81 amplifying chloramphenicol resistance gene were used for verification– pSEVA351 (empty plasmid); Y: pSEVA351-YFP; M: DNA marker (kb); PCR-: negative control (no DNA). **(C)** Fluorescence measurements of *Chroococcidiopsis* sp. Helios transformed with pSEVA351-YFP. As a control, WT and Helios isolates harboring pSEVA351 are shown. Relative YFP fluorescence in relation to OD_750nm_ was quantified. The graph shows the average of three biological replicates together with the standard deviation.

To further explore the possibilities of heterologous expression in these new *Chroococcidiopsis* isolates, we constructed the plasmid pSEVA351-YFP based on the conjugal vector pSEVA351 (Figure 9A). This plasmid contains the YFP under the constitutive synthetic promoter P_c223_ (Markley et al., 2015). The pSEVA351-YFP was transferred to Helios isolates by triparental mating. After verifying the presence of the plasmid by PCR (Figure 9B), fluorescence was measured using *Chrocococcidiopsis* isolates harboring pSEVA351 (empty plasmid) and WT as controls (Figure 9C). The reporter was constitutively expressed in all the isolates, but the result was slightly lower in *Chroococcidiopsis* sp. B11.

## 4. Discussion

The diverse microbial niche found in the solar panel is home of an extremophilic community that could hold interesting biotechnological applications. So far, the bioprospection of the solar panel microbiome has resulted in finding pigmented bacteria with high potential as antioxidants (Tanner et al., 2019) or strains such as *Arthrobacter* sp. Helios or *Exiguobacterium* sp. Helios whose polyextremophilic characteristics suggest a high biotechnological potential (Castillo-López et al., 2021; Hernández-Fernández et al., 2022). In this work, we provide the first report and characterization of solar panel culturable cyanobacterial isolates that belonging to the orders Chroococcidiopsidales and Synechococcales, the latter including members of three genera-Leptolyngbya, *Oculatella*, and *Myxacorys*. These orders are also represented in other extremophile sites, such as the small water bodies in a cold high-mountain desert of Eastern Pamir, whose cyanobacterial taxonomic profile showed the presence of Synechococcales (47.1%), Nostocales (18.6%), Oscillatoriales (15.8%), Gloeobacterales (1.2%), Chroococcales (0.92%), and Chroococcidiopsidales (0.14%) orders (Khomutovska et al., 2021). Finding the orders Chroococcidiopsidales and Synechococcales in these extreme sites suggests that they could also play a pioneering role, as suggested by Wang et al. (2020). In fact, as the primary colonizers of constrained environments, they form biofilms that could favor the formation to microbial associations as EPSs of cyanobacteria can adhere to the solid surface and functions as bioadhesives while protecting cells from physical damage (Li et al., 2001; Knowles and Castenholz, 2008; Rossi and De Philippis, 2015; Parwani et al., 2021).

From all the culturable cyanobacteria isolated from the Mediterranean solar panel, we have centered our analyses on certain culture samples that by optical microscope revealed the presence of round cells and growth as single-cells or small aggregates. The taxonomic classification and the phylogenetic analysis of 16S sequences confirmed that they were four different *Chroococcidiopsis* isolates (referred to as Helios isolates in this work). The presence of this genus in a solar panel is not surprising as several studies have shown that *Chroococcidiopsis* sp. withstands conditions that result from multiple cycles of drying and wetting and/or prolonged desiccation (Friedmann, 1993; Billi et al., 2001).

A better understanding of how different environmental factors affect the growth of the *Chroococcidiopsis* isolates in this work will provide a better insight into the potential of these isolates for further applications. In this study, we have tested how salt concentration, temperature, pH, desiccation, UV exposure, and the presence of other nitrogen/carbon sources affect the growth of Helios isolates.

Salt tolerance studies concerning members of the genus *Chroococcidiopsis* have showed that there are substantial differences among the isolates obtained from different environments, suggesting the existence of two groups: freshwater (those who fail to grow at concentrations above 250 mM of added sea salt) and salt-tolerant clades (moderate halophiles that require 250 to 500 mM of added sea salt for their optimal growth; Cumbers and Rothschild, 2014). On average, NaCl accounts for about 73% of the salt content in seawater, which corresponds to 437.5 mM (Cumbers and Rothschild, 2014). In our results (Figure 3), the *Chroococcidiopsis* isolates from the solar panel displayed a similar profile to the freshwater clade, being able to grow up to NaCl 250 mM (B11) or showing a growth sensitive to the presence of salt in the medium (B15). As growth is clearly affected by the presence of NaCl in several *Chroococcidiopsis* sp. Helios, adaptation processes of the candidate strains for their cultivation in seawater would be necessary.

pH and temperature are other important factors in the growth, establishment, and diversity of cyanobacteria. It has been generally reported that cyanobacteria prefer neutral pH for optimum growth (Nayak and Prasanna, 2007; Temraleeva et al., 2016); in fact, all the Helios isolates grow better at pH 7.5. Only the B13 isolate could tolerate pH 9.0 in liquid medium. Other cyanobacteria, such as *Leptolyngbya* sp., seem to resist higher pH levels (Taton et al., 2012). In this work, *Leptolyngbya* sp. UTEX1 was kept in a culture medium with a pH of 9.5. Additionally, Helios isolates can be classified as mesophilic as the maximum cell growth rate occurred at 30°C, with minimal survival at 40°C when growing in liquid medium. These data are consistent with those published for other *Chroococcidiopsis* spp. (Ocampo-Friedmann et al., 1988; Dillon et al., 2002). It has been reported that the presence of water reduces the upper temperature survival limit compared to desiccated conditions, probably due to protein inactivation (Cockell et al., 2017). Considering that the Helios isolates came from a solar panel, their inability to grow at 50°C in liquid media could be explained because of the lack of water in their original ecological niche. In agreement with this, several works have shown that extreme arid conditions are endured better by organisms that can dehydrate prior to being exposed to these extremes, minimizing thermal and radiation damage (Cockell et al., 2017). Nevertheless, the growth of these isolates in liquid medium at 40°C is relevant, as most industrial applications are carried out in these conditions, with the advantage that when dried, these bacteria can survive and be reused. The desiccation resistance of *Chroococcidiopsis* sp. Helios will be discussed later.

Although it has been reported that some *Chroococcidiopsis* strains may meet the demand for biologically usable nitrogen by nitrogen fixation under microaerobic laboratory conditions (Rippka et al., 1979; Stewart, 1980), other strains do not have this ability and are dependent on a supply of combined nitrogen (e.g., NO3−, NH4+ or organic nitrogen; Billi and Caiola, 1996). Helios isolates are also dependent on sources of nitrogen in the medium. On the other hand, as some of the applications of cyanobacteria lie on residual waters which may contain different sources of nitrogen, we have also studied the tolerance of the Helios isolates to other sources of nitrogen apart from nitrates, present in the BG11 growth medium. Urea, a metabolite easily found in residual waters, was not toxic for *Chroococcidiopsis* sp. Helios, even though some strains like B14 and B15, failed to grow when it was used as a sole nitrogen source. Therefore, Helios isolates need a nitrogen source to grow, and this can be obtained from nitrates, urea, or both combined. This fact is promising in terms of industrial applications because it has been suggested that the use of NPK-fertilizers (an agricultural fertilizer) as culture media would reduce production costs in large-scale cultures for cyanobacteria farming (Montero-Lobato et al., 2020).

It has been reported that several bacterial groups are able to generate extracellular compounds to establish their niche and survive, especially in arid regions. Specifically, bacteria use biofilms as a structure to resist adverse conditions (Derikvand et al., 2017; Wu et al., 2021). Extremophiles have developed mechanisms that lead to the production of valuable substances in order to survive (Banerjee et al., 2021). Part of the carbohydrates accumulate in cyanobacteria as exopolysaccharides (EPS), depending on species and cultivation conditions, and could contribute to biofilm formation, which protects cyanobacteria against ecological stresses, provides a moist environment, and favors cell aggregation (Potts, 1999; Rossi and De Philippis, 2015; Decho and Gutierrez, 2017). Regarding biotechnological applications, this shell of exopolysaccharides has potential uses in terms of commercial applications in industry [e.g., the dairy, cosmetics, research, agricultural, and petroleum industries (Rana and Upadhyay, 2020) or biomedicine (Mohd Nadzir et al., 2021)]. As part of the further characterization of *Chroococcidiopsis* sp. Helios isolates, the carbohydrate content and biofilm formation ability were evaluated (Figure 5). The carbohydrate content of the Helios isolates is around 11% for B11, B13, and B14 and approximately 5% for B15. These values are within those reported for different cyanobacteria (González-Fernández and Ballesteros, 2012) and more concretely, for *Spirulina maxima* and *Synechococcus* (13–16% dry weight, Becker, 2003) or *Synechocystis* 6803 (10% dry weight, Patel et al., 2016). On the other hand, as aforementioned, cyanobacteria are often the first colonizers, so biofilm formation provides a structure that favors the development of microbial associations and protects them against severe conditions. In this sense, it has been reported that the dried biofilm of *Chroococcidiopsis* sp. CCMEE 029 could be revived after nearly 2 years of exposure to space vacuum (Mosca et al., 2021) and that the biofilm’s status helps combat stresses (Baque et al., 2013a; Billi et al., 2019). Apart from *Chroococcidiopsis* sp. B11, all the Helios isolates were capable of forming biofilms, reinforcing the idea of *Chroococcidiopsis* sp. as one of the cyanobacteria pioneering colonizers in the solar panel and a mechanism that would help these extremophiles to survive in such a harsh environment.

The solar panels in Valencia are exposed to radiation and low water availability with an optimized inclination to maximize insolation and without any source of substrate to attenuate radiation. Consequently, we expected to isolate cyanobacteria that were resistant to desiccation and UV-C exposure, as our data has confirmed. In fact, a marked feature of *Chroococcidiopsis* is its resistance to desiccation and irradiation, given that it is able to protect itself by living an endolithic mode of life, a few millimeters beneath the surface or on the underside of rocks, living mainly on nutrients brought in by wind and rain (Bothe, 2019).

Our results suggest that Helios isolates could provide a good model to study the desiccation process, as they can be revived after a year in harsh conditions (Figure 6). Broader knowledge concerning how these isolates could reach xerotolerance may open doors to biotechnological applications, from plant-associated bacteria that could be an alternative for fertilizers and pesticides to industrial uses (Garcia, 2011; Greffe and Michiels, 2020).

Regarding UV exposure, cyanobacteria are generally more resistant to UV radiation than many microorganisms, since they live in habitats where sunlight is a constant, so they usually possess mechanisms to avoid or counteract the damage produced. In fact, several strains of *Chroococcidiopsis* sp. have been shown to resist UV-C. On the other hand, a model bacterium for radiation studies because of its resistance is Deinococcus radiodurans, an extremophile commonly isolated from habitats exposed to periodic desiccation, for instance soil or strong environmental UV radiation as the high atmosphere (Krisko and Radman, 2013; Gerber et al., 2015; Jin et al., 2019; Bruckbauer and Cox, 2021). In this work, we evaluated the resistance of Helios isolates to UV-C exposure up to 1,000 J m^−2^ in liquid media, as most applications of extremophiles still depend on water solutions. The higher resistance to UV-C radiation found in the Helios isolates compared with *E. coli* was similar to what is described for *Chroococcidiopsis* sp. CCMEE 029 (Baque et al., 2013b) Recently, the repair robustness of DNA lesions accumulated under Mars-like conditions was demonstrated and an overexpression of DNA repair genes has also been reported in this genus (Mosca et al., 2019; Napoli et al., 2022), mechanisms that could contribute to resilience and radiation-tolerance of this microorganism.

Finally, the use of *Chroococcidiopsis* as a biotechnological strain or even as a production platform requires the development of genetic toolboxes specific for them. pDU1 replicon of *Nostoc* sp. PCC 7524 was successfully transferred to 3 out of 5 isolates of *Chroococcidiopsis via* conjugation or electroporation (Billi et al., 2001). In this study, we evaluated the genetic transformation of Helios isolates using four SEVA plasmids harboring different replicons: the RSF1010, the RK2 replicon, the pBBR1, and the pRO1600/ColE origins (a hybrid of the pRO1600 origin and the ColE1 replication sequence). Most cyanobacteria admit replicative plasmids with the RSF1010 origin. The RK2 replication origin can replicate autonomously in *Synechocystis* but not pBBR1 or pRO1600/ColE origin (Vasudevan et al., 2019). In our research, it was possible to transform Helios isolates with all the four replicons tried, expanding the replication origins available for this cyanobacterium. In other *Chroococcidiopsis* sp. strains, the failure to obtain transconjugants was suggested to be due to the presence of genetic barriers, such as host-specific restriction endonucleases (Billi et al., 2001). However, in the Helios isolates presented here, the protection against foreign DNA by restriction systems did not appear to be a significant barrier for genetic manipulation since conjugation was possible, which stands in opposition to what has been reported for other strains like *Anabaena* sp. strain PCC 7120 (Elhai et al., 1997).

In addition to the transformation results obtained, the transfer of a plasmid with the CRISPR-Cas12a technology (Figure 8) as well as the expression of YFP (Figure 9), together with the fact that the Helios isolates are extremophilic microorganisms, opens the door to their future study for astrobiological and biotechnological purposes. The possibility of genomic editing through CRISPR could help us to understand the mechanisms involved in the resistance of these cyanobacteria or to develop higher tolerant strains. What is more, applying these genetic tools can lead to strain modifications for improved growth properties, and to the production of desired molecules, especially pigments like scytonemin, which has an important commercial value in cosmetics and medicine (Gao et al., 2021).

## 5. Conclusion

In this work we have identified and characterized several *Chroococcidiopsis* isolates from a solar panel of a Mediterranean city. The findings of this genus in this extreme habitat reinforce its role as a pioneering colonizer strain. The study of the physicochemical parameters of the isolated isolates has shown us that they can resist irradiation up to 1,000 J m^−2^ and desiccation for long periods, being able to restore growth after 1 year. On the other hand, we have also shown that these *Chroococcidiopsis* isolates can be transformed with SEVA vectors, including one that harbors the Cpf1 nuclease, widely used in CRISPR-mediated editing processes. The capability to be genetically modified opens the door to new perspectives for these extremophile isolates, for instance their use in biotechnological applications or in astrobiology.

## Data availability statement

The original contributions presented in the study are included in the article/Supplementary material, further inquiries can be directed to the corresponding authors.

## Author contributions

SB, GG, and JN contributed equally to the conception and design of the study, analysed and discussed the data, wrote the draft of the manuscript, and made the revisions. AB-R performed the initial screening and isolation of cyanobacteria from the solar panel. SB, RA, and GG conducted the characterization and studies on the bacterial isolates. All authors read and approved the submitted version.

## Funding

This research was supported by grants ALGATEC-CM (P2018/BAA-4532) co-financed by the European Social Fund and the European Regional Development Fund, Seth (RTI2018-095584-B-C41-42-43-44) from the Ministry of Science and Innovation of Spain and Helios (BIO2015-66960-C3-3-R) from the Ministry of Economy and Competitiveness of Spain. SB was supported by a fellowship from “Universidad Complutense de Madrid” (Spain).

## Conflict of interest

The authors declare that the research was conducted in the absence of any commercial or financial relationships that could be construed as a potential conflict of interest.

## Publisher’s note

All claims expressed in this article are solely those of the authors and do not necessarily represent those of their affiliated organizations, or those of the publisher, the editors and the reviewers. Any product that may be evaluated in this article, or claim that may be made by its manufacturer, is not guaranteed or endorsed by the publisher.

## Footnotes

^1^ http://blast.ncbi.nlm.nih.gov
