# Supplementary material for: First characterization of cultivable extremophile Chroococcidiopsis isolates from a solar panel
Source: Front Microbiol. 2023 Feb 17;14:982422. doi: 10.3389/fmicb.2023.982422 (PMC9982165; doi:10.3389/fmicb.2023.982422)
Supplement: Supplementary file 1 [file Table_1.DOCX]

**Table S1. Composition of culture medium used for cyanobacteria isolation**

|  | UTEX | | BG11 | Castenholz-D | | MDM |
| --- | --- | --- | --- | --- | --- | --- |
| Macronutrients (g/L) | |  | | |  | |
| NaHCO_3_ | 13.61 | | - | - | | - |
| Na_2_HPO_4_ | - | | - | 0.11 | | - |
| Na_2_CO_3_ | 4.03 | | 0.02 |  | | - |
| NaNO_3_ * | 2.50 | | 1.50 | 0.70 | | - |
| NaCl | 1.00 | | - | 0.008 | | 0.01 |
| K_2_HPO_4_ | 0.50 | | 0.03 | - | | 0.025 |
| K_2_SO_4_ | 1.00 | | - | - | | - |
| KNO_3_ * | - | | - | 0.10 | | - |
| MgSO_4_ * 7 H_2_O | 0.20 | | 0.075 | 0.10 | | 0.025 |
| CaCl_2_ * 2 H_2_O | 0.04 | | 0.036 | - | | 0.001 |
| CaSO_4_ * 2 H_2_O | - | | - | 0.06 | | - |
| Nitrilotriacetic acid | - | | - | 0.10 | | - |
| Micronutrients (mg/L) | |  | | |  | |
| Vitamin B12 | 0.135 | | - | - | | - |
| Na_2_EDTA * 2 H_2_O | 4.550 | | 1.00 | - | | - |
| FeCl_3_ | 0.350 | | - | 2.28 | | - |
| FeSO_4_ * 7 H_2_O | - | | - | - | | 0.0002 |
| MnCl_2_* 4 H_2_O | 0.258 | | 1.81 | - | | - |
| MnSO_4_ * H2O | - | | - | 11.4 | | 0.25 |
| ZnCl_2_ | 0.030 | | - | - | | - |
| CoCl_2_* 6 H_2_O | 0.024 | | 0.050 | 0.0125 | | - |
| Na_2_MoO_4_*H_2_O | 0.036 | | 0.039 | 0.0125 | | 0.002 |
| CuSO_4_* 5 H_2_O | 0.020 | | 0.080 | 0.0125 | | 0.008 |
| ZnSO_4_ * 7 H_2_O | 0.044 | | 0.220 | 0.25 | | 0.022 |
| H_3_BO_3_ | 0.620 | | 2.86 | 0.25 | | 0.286 |
| H_2_SO_4_ | - | | - | 0.5 μL | | - |
| Citric acid | - | | 6 | - | | - |
| Ammonium ferric citrate | - | | 6 | - | | - |
| * For BG11_0_ and MDM_0_ (medium without combined nitrogen sources), these components are omitted. | | | | | | |
