# Supplementary material for: First characterization of cultivable extremophile Chroococcidiopsis isolates from a solar panel
Source: Front Microbiol. 2023 Feb 17;14:982422. doi: 10.3389/fmicb.2023.982422 (PMC9982165; doi:10.3389/fmicb.2023.982422)
Supplement: Supplementary file 2 [file Table_2.docx]

**Table S2. PCR primers used in this study**

| Primer | Sequence (5´- 3´) | PCR | Reference |
| --- | --- | --- | --- |
| Cyanobacteria identification, 16S rRNA amplification | | | |
| CYA106-Fw | CGGACGGG GAGTAACGCGTGA | Tm 55ºC, 30 s, 72ºC, 90 s | (Nübel et al., 1997) |
| CYA359-Fw | GGGGAATYTTCCGCAATGGG |  | (Nübel et al., 1997) |
| CYA781-Rv(a) | GACTACTGGGGTATCTAATCCCAT |  | (Nübel et al., 1997) |
| CYA781-Rv(b) | GACTACAGGGGTATCTAATCCCTTT |  | (Nübel et al., 1997) |
| Identification of eukaryotic microorganisms, 18S rRNA amplification | | | |
| ss5-Fw | GGTGATCCTGCCAGTAGTCATATGCTTG | Tm 55°C, 30 s, 72°C, 120 s, | (Matsumoto et al., 2010) |
| ss3-Rv | GATCCTTCCGCAGGTTCACCTACGGAAACC |  |  |
| 18S-Fw | GTCAGAGGTGAAATTCTTGGATTTA | Tm 55°C, 30 s, 72°C, 120 s, | (Gross et al., 2001) |
| 18S-Rv | AGGGCAGGGACGTAATCAACG |  |  |
| Conjugation checking, CmR cassette amplification | | | |
| R24 | AGCGGATAACAATTTCACACAGGA | Tm 55°C,30 s, 72°C, 120 s, | This work |
| R24rv | TCATGGTCATAGCTGTTTCC |  | This work |
| Oligo8 | TTACGCCCCGCCCTGCCACT |  | This work |
| SB80 | ATAAGATCACTACCGGGCGT | Tm 57°C, 30 s, 72°C, 30 s, | This work |
| SB81 | GTGTAGAAACTGCCGGAAATC |  | This work |
| Construction of pSEVA351-YFP, cassette amplification | | | |
| SB80 | AGATGCCACGTGCAAATAGCTAGCTCACTCGGTC | Tm 57°C, 30 s, 72°C, 120 s, | This work |
| SB81 | GCAAACGAAGACAGTCGACTGCAGCAGACTCATTAACATACGCT |  | This work |

Forward primers CYA106F and CYA359F were used in alternative reactions.

Y, a C/T nucleotide degeneracy.

Reverse primer CYA781R was an equimolar mixture of CYA781R(a) and CYA781R(b).

References:

Gross, W., Heilmann, I., Lenze, D., and Schnarrenberger, C. (2001). Biogeography of the Cyanidiaceae (Rhodophyta) based on 18S ribosomal RNA sequence data. Europ. J. Phycol. 36(3), 275-280. doi: 10.1080/09670260110001735428.

Matsumoto, M., Sugiyama, H., Maeda, Y., Sato, R., Tanaka, T., and Matsunaga, T. (2010). Marine diatom, Navicula sp. strain JPCC DA0580 and marine green alga, Chlorella sp. strain NKG400014 as potential sources for biodiesel production. Appl. Biochem. Biotechnol. 161(1-8), 483-490. doi: 10.1007/s12010-009-8766-x.

Nübel, U., Garcia-Pichel, F., and Muyzer, G. (1997). PCR primers to amplify 16S rRNA genes from cyanobacteria. Appl. environ. microbiol. 63(8), 3327-3332.
