# Supplementary material for: First characterization of cultivable extremophile Chroococcidiopsis isolates from a solar panel
Source: Front Microbiol. 2023 Feb 17;14:982422. doi: 10.3389/fmicb.2023.982422 (PMC9982165; doi:10.3389/fmicb.2023.982422)
Supplement: Supplementary file 3 [file Table_3.docx]

**Table S3. Ecological and geographical origin of the cyanobacteria best BLAST hits obtained by querying the 16S rRNA.**

| **Blast hit** | **Location** | **Site description** | **Reference** |
| --- | --- | --- | --- |
| *Oculatella* *coburnii* WJT66-NPBG6A | Joshua Tree National Park, Mojave Desert, California, USA | Well-developed biological soil crust with algae, lichens, and mosses. Full sunlight and desert soil | (Osorio-Santos et al., 2014) |
| *Oculatella mojaviensis* CMT-3BRIN-NPC87 | Mojave Desert National Preserve, California, USA | Well-developed biological soil crust with algae, lichens, and mosses. Full sunlight and desert soil | (Osorio-Santos et al., 2014) |
| *Oculatella* *ucrainica* KZ-5-4-1 | Coast of the Sea of Azov, Kazantip Nature Reserve, spit of Aqtash Lake, Ucrania | Coquina beach, 10 m from the water's edge, cyanobacterial-algal crusts | (Mikhailyuk et al., 2016) |
| Cyanobacterium WJT24-NPBG12B / *Myxacorys* *californica* WJT24-NPBG12B | Joshua Tree National Park, California, USA. | Sandy, gravelly soil from granitic outcrop, plants and well-developed algal crusts. | (Pietrasiak et al., 2019) |
| *Pseudophormidium* sp. FI6-MK20 / *Myxacorys californica* FI6-MK20 | Fort Irwin | Undisturbed *Larrea* and *Ephedra* community in dry wash with granitic soils | (Johansen et al., 2001) |
| *Pseudophormidium* sp. CMT-1FSIN-NPC23/ *Myxacorys californica* CMT-1FSIN-NPC23 | Mojave National Preserve, USA | Sandy, gravelly soils with some biocrusts, plants rare | (Pietrasiak et al., 2019) |
| *Leptolyngbya* sp. RV74 | Unavailable | Unavailable | Unpublished |
| *Chroococcidiopsis* sp. CENA240 | Fresh water, Cistern Canindé, Ceara, Brazil | Caatinga, semiarid region | (Costa et al., 2016) |
| *Chroococcidiopsis* sp. CENA246 | Fresh water, Cistern Canindé, Ceara, Brazil | Caatinga, semiarid region | (Costa et al., 2016) |
| *Chroococcidiopsis* sp. CCNUC1 | Central China Normal University (CCNU), China | Arid and humid moss on forest limestone | (Zhang et al., 2019) |
| *Chroococcidiopsis* sp. CCNUC3 | Central China Normal University (CCNU), China | Arid moss on forest limestone | (Zhang et al., 2019) |
| *Chroococcidiopsis* sp. SAG 2025 | Ewa Plain, Hawaii | Endolithic from a coral rock | (Friedl and Schlösser, 2000) |
| *Chroococcidiopsis* sp. YRS 4a | Sri Lanka | Unavailable | (Wanigatunge et al., 2014) |

**References:**

Costa, N.B., Kolman, M.A., and Giani, A. (2016). Cyanobacteria diversity in alkaline saline lakes in the Brazilian Pantanal wetland: A polyphasic approach. *J. Plankton Res.* 38(6), 1389-1403.

Friedl, T., and Schlösser, U.G. (2000). Additions to the culture collection of algae at Göttingen since 1997. *Nova Hedwigia* 71, 243–262.

Johansen, J.R., Britton, C., Rosati, T.C., Li, X., St. Clair, L.L., Webb, B.L., et al. (2001). Microbiotic crust of the Mojave Desert: factors influencing distribution and abundance. *Proc. Int. Conf*. 123, 341–371.

Mikhailyuk, T.I., Vinogradova, O.N., Glaser, K., and Karsten, U. (2016). New taxa for the flora of Ukraine, in the context of modern approaches to taxonomy of Cyanoprokaryota/Cyanobacteria. *Int. J. Algae* 18(4), 301-320. doi: 10.1615/InterJAlgae.v18.i4.10.

Osorio-Santos, K., Pietrasiak, N., Bohunická, M., Miscoe, L.H., Kováčik, L., Martin, M.P., et al. (2014). Seven new species of *Oculatella* (Pseudanabaenales, Cyanobacteria): Taxonomically recognizing cryptic diversification. *Europ.* *J. phycol.* 49(4), 450-470.

Pietrasiak, N., Osorio‐Santos, K., Shalygin, S., Martin, M.P., and Johansen, J.R. (2019). When is a lineage a species? A case study in *Myxacorys* gen. nov. (Synechococcales: Cyanobacteria) with the description of two new species from the Americas. *J. phycol*. 55(9), 976-996.

Wanigatunge, R.P., Magana-Arachchi, D.N., Chandrasekharan, N.V., and Kulasooriya, S.A. (2014). Genetic diversity and molecular phylogeny of cyanobacteria from Sri Lanka based on 16S rRNA gene. *Environ. Eng. Res*. 19(4), 317-329. doi: 10.4491/eer.2014.035.

Zhang, Z.C., Li, Z.K., Yin, Y.C., Li, Y., Jia, Y., Chen, M., et al. (2019). Widespread occurrence and unexpected diversity of red-shifted chlorophyll producing cyanobacteria in humid subtropical forest ecosystems. *Environ. Microbiol*. 21(4), 1497-1510. doi: 10.1111/1462-2920.14582.
