# Supplementary material for: First characterization of cultivable extremophile Chroococcidiopsis isolates from a solar panel
Source: Front Microbiol. 2023 Feb 17;14:982422. doi: 10.3389/fmicb.2023.982422 (PMC9982165; doi:10.3389/fmicb.2023.982422)
Supplement: Supplementary file 4 [file Table_4.docx]

**Table S4. Growth parameters of *Chroococcidiopsis* isolates**

| **Isolate** | **Doubling time (days)** | **Relation OD750nm and cells/mL** |
| --- | --- | --- |
| *Chroococcidiopsis* sp. B11 | 6.66 ± 1.55 | cells/mL = 8.199*106 OD750nm; R2 = 0.95 |
| *Chroococcidiopsis* sp. B13 | 3.24 ± 0.30 | cells/mL = 1.017*107 OD750nm; R2 = 0.97 |
| *Chroococcidiopsis* sp. B14 | 3.32 ± 0.30 | cells/mL = 4.025*106 OD750nm; R2 = 0.93 |
| *Chroococcidiopsis* sp. B15 | 2.39 ± 0.21 | cells/mL = 1.058*107 OD750nm; R2 = 0.90 |
