# Supplementary material for: First characterization of cultivable extremophile Chroococcidiopsis isolates from a solar panel
Source: Front Microbiol. 2023 Feb 17;14:982422. doi: 10.3389/fmicb.2023.982422 (PMC9982165; doi:10.3389/fmicb.2023.982422)
Supplement: Supplementary file 13 [file Table_13.DOCX]

Table S13. Antibiotic sensitivity of *Chroococcidiopsis* isolates

|  | Km | | | | Cm | | | | Spt | | | | Spm | | | |
| --- | --- | --- | --- | --- | --- | --- | --- | --- | --- | --- | --- | --- | --- | --- | --- | --- |
| Strain | 20 | 50 | 75 | 100 | 7.5 | 15 | 30 | 75 | 2 | 4 | 8 | 20 | 2 | 4 | 8 | 20 |
| B11 | **+** | **+** | **+** | **+** | - | - | - | - | + | + | + | + | - | - | - | - |
| B13 | **+** | **+** | **+** | **+** | - | - | - | - | + | + | + | + | - | - | - | - |
| B14 | **+** | **+** | **+** | **-** | - | - | - | - | + | + | + | + | - | - | - | - |
| B15 | **+** | **+** | **+** | **-** | - | - | - | - | + | + | + | + | - | - | - | - |
|  |  |  |  |  |  |  |  |  |  |  |  |  |  |  |  |  |
|  | **Em** | | | | **Gm** | | | | **Nal** | | | | **Nm** | | | |
| Strain | 20 | 40 | 80 | 200 | 2 | 4 | 8 | 20 | 7.5 | 15 | 30 | 75 | 25 | 50 | 100 | 250 |
| B11 | - | - | - | - | + | + | + | + | + | + | + | - | + | - | - | - |
| B13 | - | - | - | - | + | + | + | + | + | + | + | - | + | - | - | - |
| B14 | - | - | - | - | + | + | + | + | + | + | +/- | - | + | - | - | - |
| B15 | - | - | - | - | + | + | + | + | + | + | + | - | + | - | - | - |

The concentrations shown for each antibiotic correspond to μg/mL. +: robust growth; +/-: some growth; -: no growth. Antibiotics: kanamycin (Km), chloramphenicol (Cm), spectinomycin (Spt), streptomycin (Spm), erythromycin (Em), gentamicin (Gm), nalidixic acid (Nal), and neomycin (Nm).
