# Supplementary figures and images for: First characterization of cultivable extremophile Chroococcidiopsis isolates from a solar panel
Source: Front Microbiol. 2023 Feb 17;14:982422. doi: 10.3389/fmicb.2023.982422 (PMC9982165; doi:10.3389/fmicb.2023.982422)

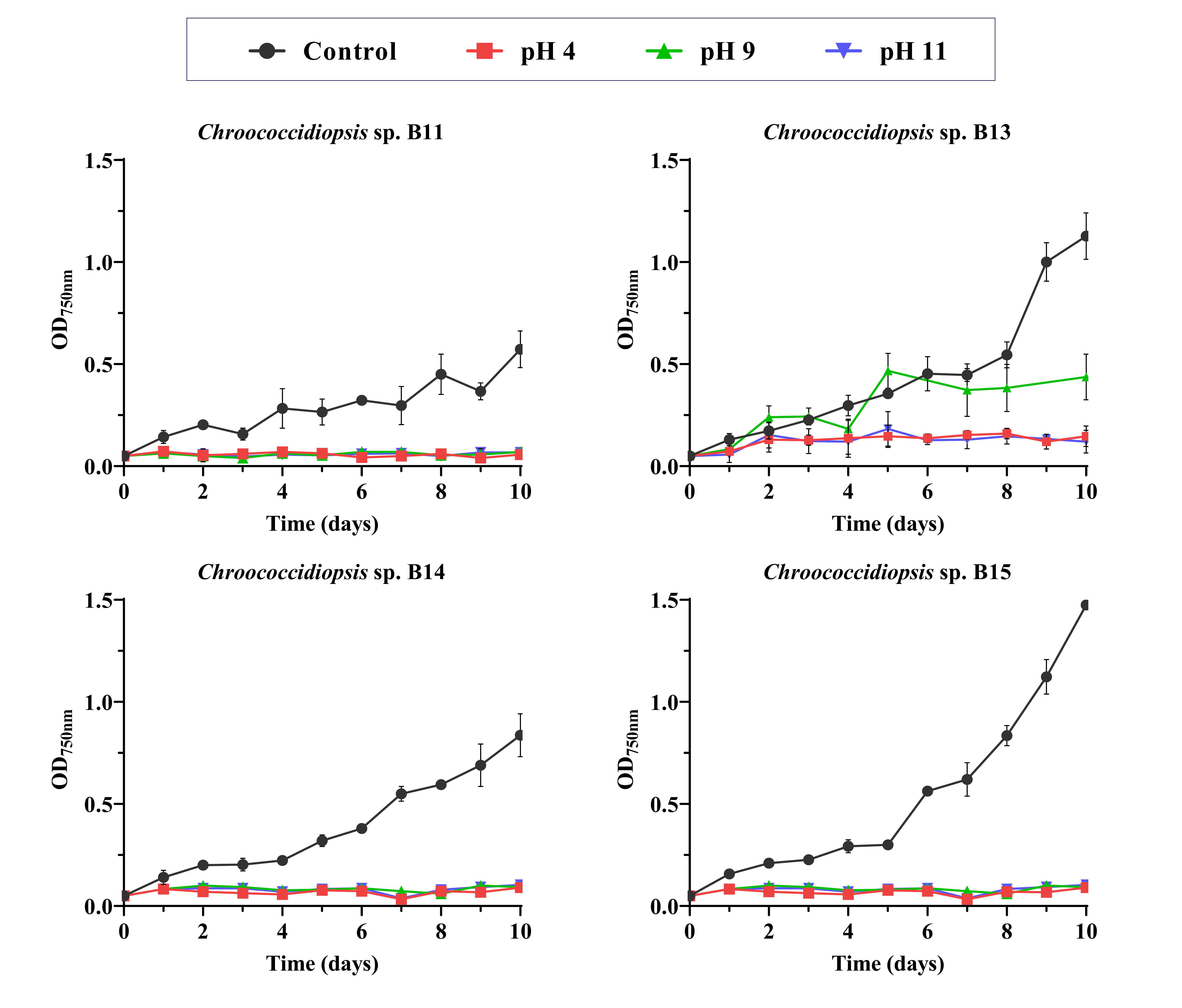

Supplement: Supplementary file 14 [file Image_1.JPEG]

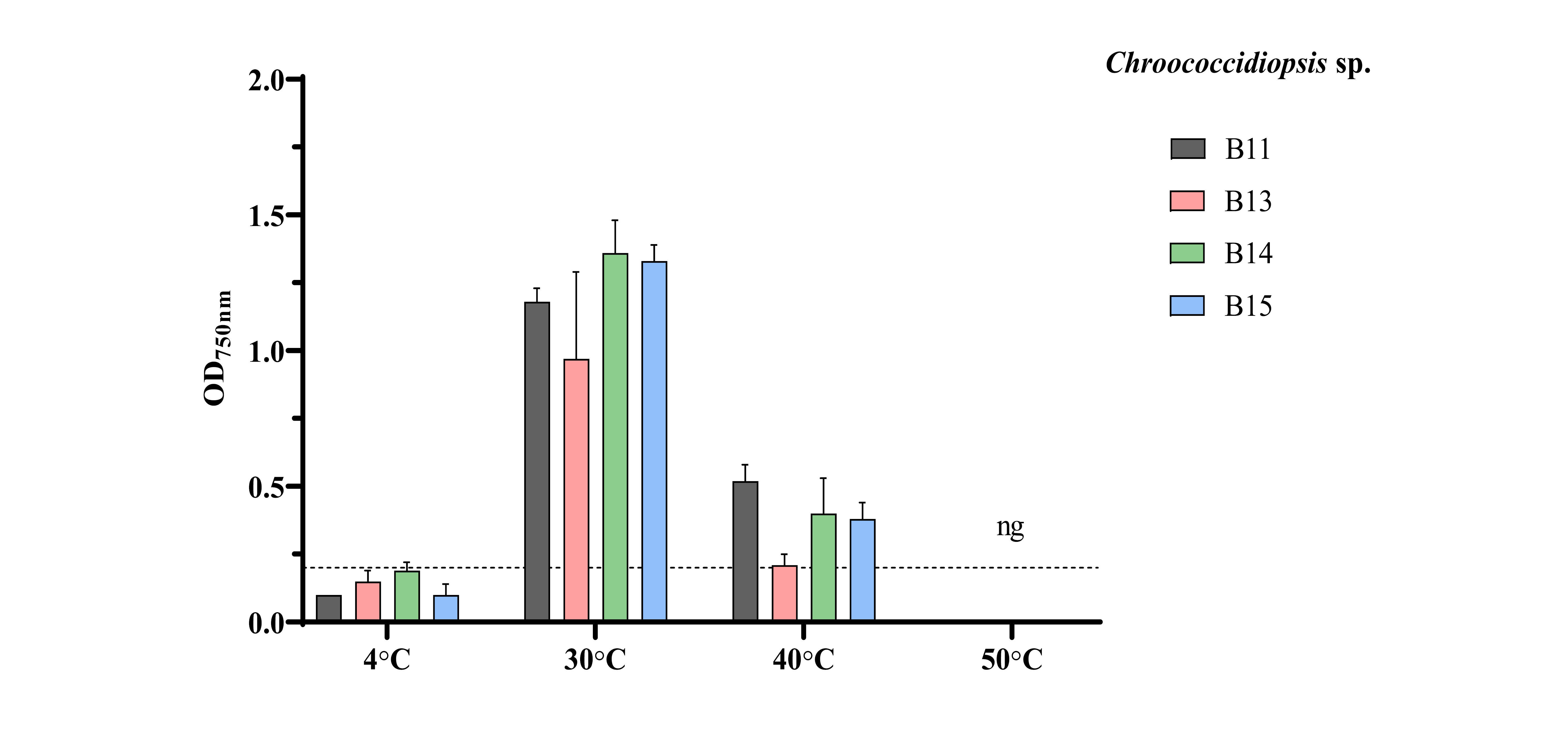

Supplement: Supplementary file 15 [file Image_2.JPEG]

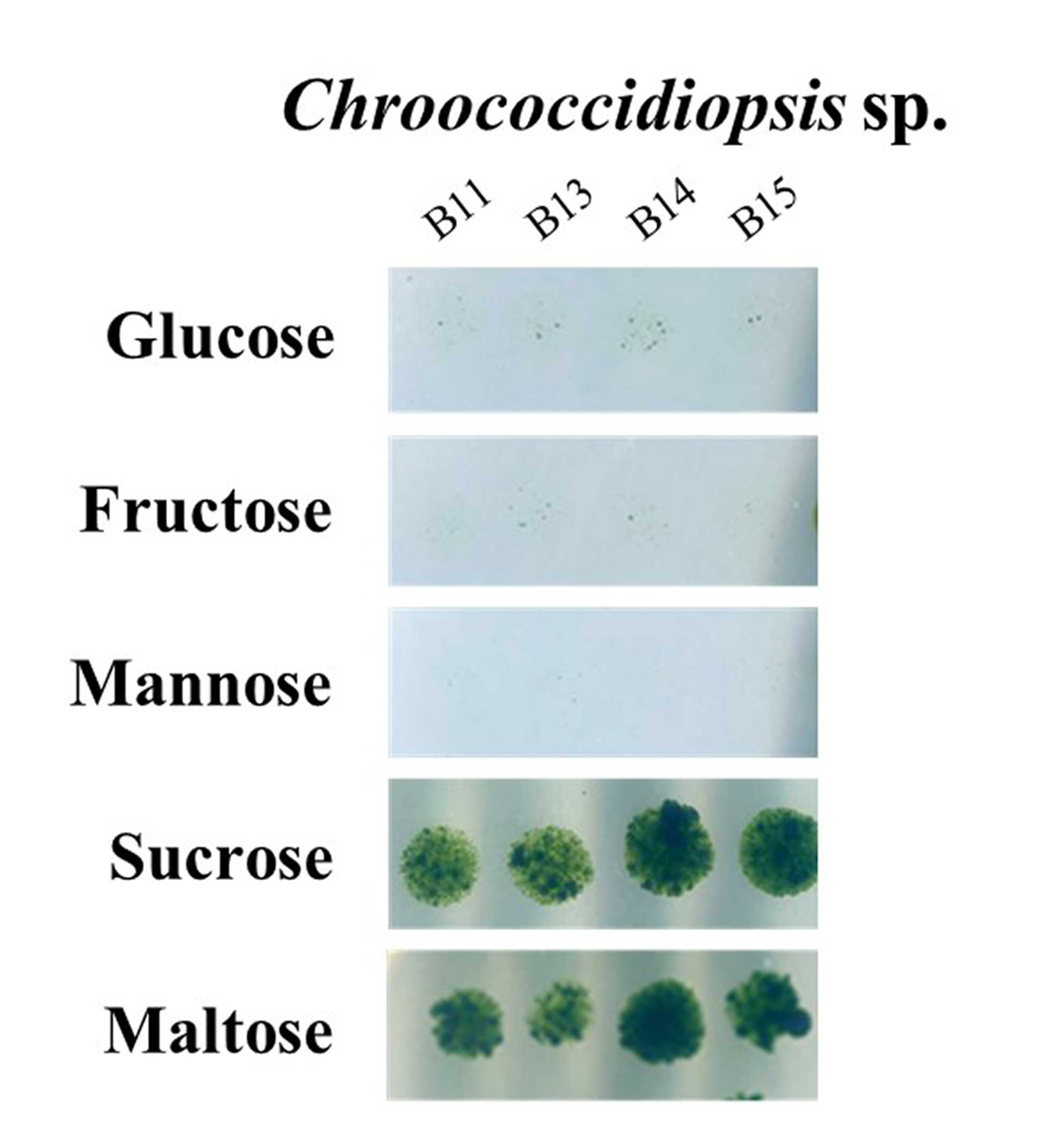

Supplement: Supplementary file 16 [file Image_3.JPEG]

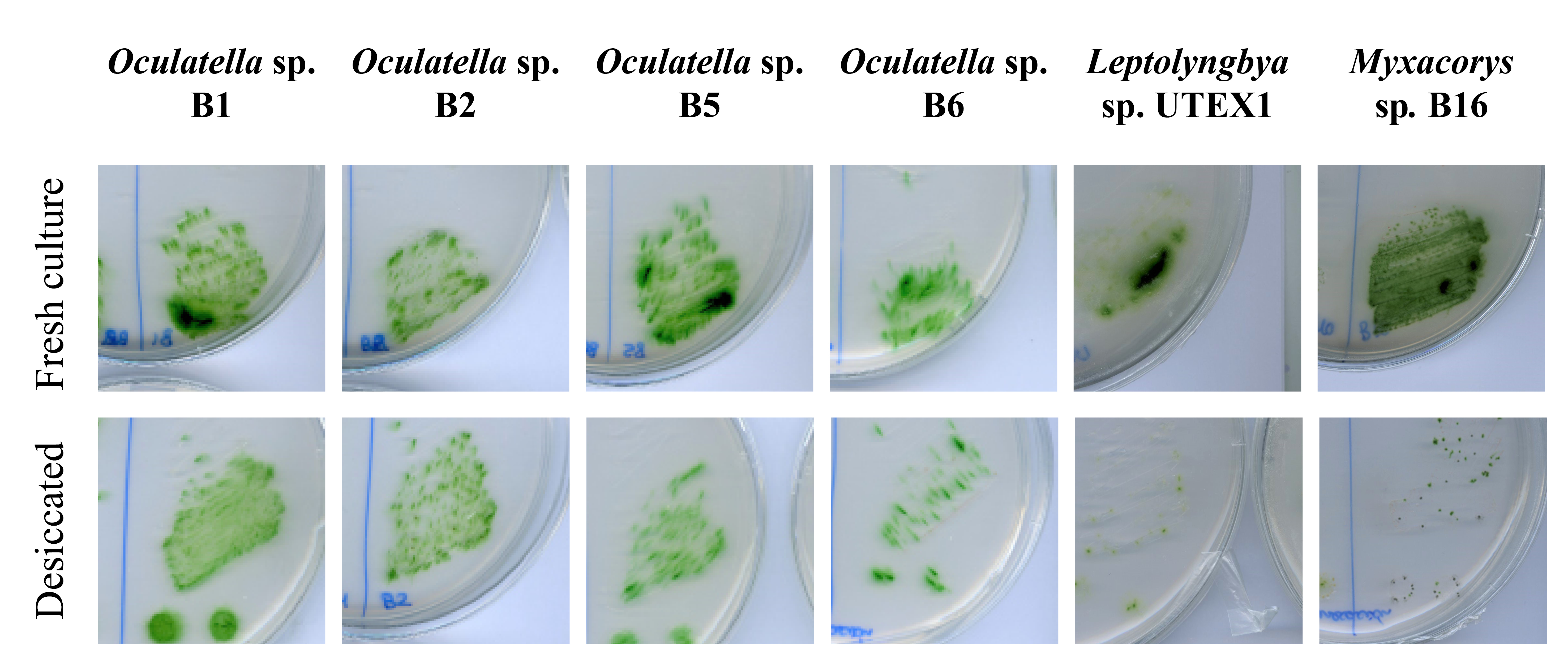

Supplement: Supplementary file 17 [file Image_4.JPEG]

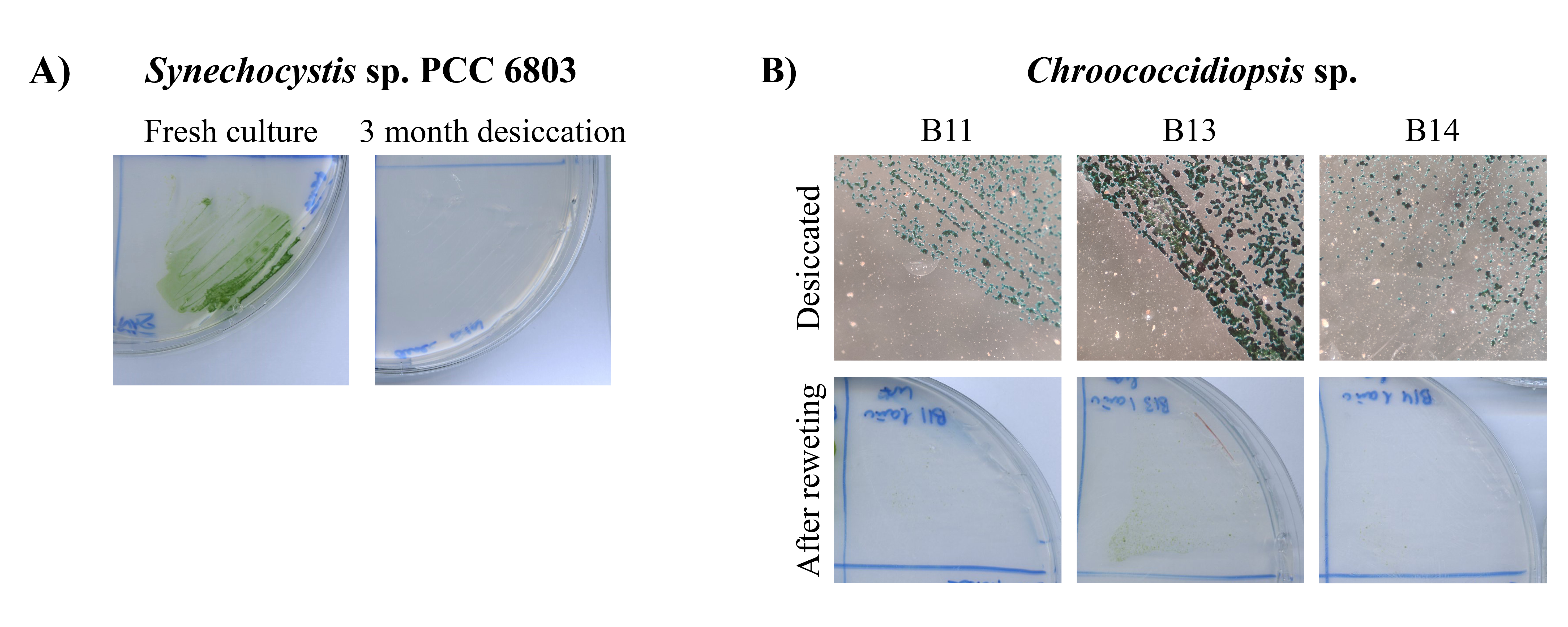

Supplement: Supplementary file 18 [file Image_5.JPEG]
